# RBM39 shapes innate immunity by controlling the expression of key factors of the interferon response

**DOI:** 10.3389/fimmu.2025.1568056

**Published:** 2025-04-22

**Authors:** Teng-Feng Li, Paul Rothhaar, Arthur Lang, Oliver Grünvogel, Ombretta Colasanti, Santa Mariela Olivera Ugarte, Jannik Traut, Antonio Piras, Nelson Acosta-Rivero, Vladimir Gonçalves Magalhães, Emely Springer, Andreas Betz, Hao-En Huang, Jeongbin Park, Ruiyue Qiu, Gnimah Eva Gnouamozi, Ann-Kathrin Mehnert, Viet Loan Dao Thi, Stephan Urban, Martina Muckenthaler, Matthias Schlesner, Dirk Wohlleber, Marco Binder, Ralf Bartenschlager, Andreas Pichlmair, Volker Lohmann

**Affiliations:** 1 Department of Infectious Diseases, Molecular Virology, Section Virus-Host-Interactions, Medical Faculty Heidelberg, Heidelberg University, Heidelberg, Germany; 2 Institute of Virology, School of Medicine, Technical University of Munich, Munich, Germany; 3 Department of Infectious Diseases, Molecular Virology, Medical Faculty Heidelberg, Heidelberg University, Heidelberg, Germany; 4 Division of Virus-Associated Carcinogenesis, German Cancer Research Center (DKFZ), Heidelberg, Germany; 5 Institute of Molecular Immunology, University Hospital Klinikum rechts der Isar, Technical University of Munich, Munich, Germany; 6 Bioinformatics and Omics Data Analysis, German Cancer Research Center (DKFZ), Heidelberg, Germany; 7 Faculty of Biosciences, Heidelberg University, Heidelberg, Germany; 8 School of Biomedical Convergence Engineering, Pusan National University, Yangsan, Republic of Korea; 9 Heidelberg University, Medical Faculty, Department of Pediatric Oncology, Hematology, Immunology and Pneumology, Heidelberg, Germany; 10 Department of Infectious Diseases, Virology, Medical Faculty Heidelberg, Heidelberg University, Heidelberg, Germany; 11 German Center for Infection Research (DZIF), Heidelberg Partner Site, Heidelberg, Germany; 12 Biomedical Informatics, Data Mining and Data Analytics, Faculty of Applied Computer Science and Medical Faculty, University of Augsburg, Augsburg, Germany; 13 German Center for Infection Research (DZIF), Munich Partner Site, Munich, Germany

**Keywords:** RBM39, IRF3, STAT1, STAT2, IFNs, splicing

## Abstract

**Background and aims:**

The contribution of innate immunity to clearance of viral infections of the liver, in particular sensing via Toll-like receptor 3 (TLR3), is incompletely understood. We aimed to identify the factors contributing to the TLR3 response in hepatocytes via CRISPR/Cas9 screening.

**Methods:**

A genome-wide CRISPR/Cas9 screen on the TLR3 pathway was performed in two liver-derived cell lines, followed by siRNA knockdown validation. SiRNA knockdown and indisulam treatment were used to study the role of RNA-binding motif protein 39 (RBM39) in innate immunity upon poly(I:C) or cytokine treatment and viral infections. Transcriptome, proteome, and alternative splicing were studied via RNA sequencing and mass spectrometry upon depletion of RBM39.

**Results:**

Our CRISPR/Cas9 screen identified RBM39, which is highly expressed in hepatocytes, as an important regulator of the TLR3 pathway. Knockdown of RBM39 or treatment with indisulam, an aryl sulfonamide drug targeting RBM39 for proteasomal degradation, strongly reduced the induction of interferon-stimulated genes (ISGs) in response to double-stranded RNA (dsRNA) or viral infections. RNA sequencing (seq) and mass spectrometry identified that transcription and/or splicing of the key pathway components IRF3, RIG-I, and MDA5 were affected by RBM39 depletion, along with multiple other cellular processes identified previously. RBM39 knockdown further restrained type I and type III IFN pathways by reducing the expression of individual receptor subunits and STAT1/2. The function of RBM39 was furthermore not restricted to hepatocytes.

**Conclusion:**

We identified RBM39 as a regulatory factor of cell intrinsic innate immune signaling. Depletion of RBM39 impaired TLR3, RIG-I/MDA5, and IFN responses by affecting the basal expression of key pathway components.

## Introduction

The cell intrinsic innate immune system belongs to the first line of host defense mechanisms protecting against invading pathogens. When infected, a limited number of host pattern recognition receptors (PRRs) play a vital role in recognizing several pathogen-associated molecular patterns (PAMPs), which activate innate immune responses through complex signaling cascades to eliminate infections. Viral components, including viral DNA (1, 2), single-stranded RNA (ssRNA) (3, 4), double-stranded RNA (dsRNA) (5, 6), and virus-encoded proteins (7), are sensed by distinct PRRs. The recognition triggers the cell intrinsic innate immune response in infected cells. For instance, dsRNA, a replication intermediate of many RNA viruses, is sensed by cytosolic retinoic acid-inducible gene (RIG)-I-like receptors [RLRs, RIG-I, and melanoma differentiation-associated gene 5 (MDA5)] (5, 6, 8) and endosomal Toll-like receptor 3 (TLR3) (9). These PRRs then recruit the adaptor proteins mitochondrial antiviral signaling protein (MAVS) and TIR domain containing adaptor molecule 1 (TRIF), respectively, activating a chain of kinases, E3 ubiquitin ligases, and E2 ubiquitin-conjugating enzymes. This leads to nuclear factor kappa B subunit 1 (NF-κB) pathway activation and to the production of pro-inflammatory cytokines or phosphorylation of IRF3, leading to its dimerization and translocation from the cytoplasm into the nucleus. Subsequently, IRF3 binds to responsive promoters and induces the transcription of type I/type III interferons (IFNs) and interferon-stimulated genes (ISGs). Type I and type III IFNs act in an auto- and paracrine manner, binding to their receptors, interferon alpha and beta receptor (IFNAR), and interferon lambda receptor (IFNLR), on the cell surface, respectively, but signaling via a common cascade upon phosphorylation of signal transducer and activator of transcription 1 and 2 (STAT1 and STAT2). Eventually, STATs form hetero- or homodimers and translocate into the nucleus to accelerate or initiate ISG expression in infected and neighboring cells, respectively.

RNA-binding motif protein 39 (RBM39) (also known as HCC1.4 or CAPERα) was initially identified as an autoantigen in a hepatocellular carcinoma patient (10) and has been found to be upregulated in multiple cancer cells, including liver cancer (11–14). It shares high similarity with the splicing factor U2AF 65-kDa subunit (U2AF65) and contains an N-terminal serine/arginine (SR)-rich domain followed by two RNA-recognition motifs (RRM) and a third C-terminal noncanonical RRM belonging to the U2AF homology motif (UHM) family, which mediates the interaction with the UHM-ligand motif (ULM) of U2AF65 (15). Previous studies have demonstrated that RBM39 functions as a coactivator of activating protein-1 (AP-1), estrogen receptor 1 (ERα), and estrogen Receptor 2 (ERβ) (16–18) and that it is involved in multiple biological processes, including cell cycle control (19, 20), cell metabolism (20), and tumorigenesis (21, 22), possibly through its splicing activity. In recent years, aryl sulfonamide drugs, such as indisulam and E7820, were found to degrade RBM39 in a DDB1 and CUL4 associated factor 15 (DCAF15)-dependent manner and thus have been widely investigated in anticancer research (23–25). While most studies on RBM39 so far focused on cancer cells, there is limited knowledge of its function in immunity, with one *in vivo* study suggesting that indisulam-mediated modulation of RBM39 enhances anti-tumor immunity (26).

In our study, RBM39, a factor highly expressed in the liver (17), emerged as a prominent candidate through a CRISPR/Cas9-mediated screening in two liver-based cell lines aimed at identifying novel factors associated with the TLR3 response in hepatocytes. Our study reveals that RBM39 exerts a broader influence on innate immunity by modulating the transcription and splicing processes involved in the basal expression of IRF3 and other crucial factors in the IFN signaling pathway. Based on the findings, it is plausible to consider that drugs targeting RBM39, such as indisulam, may function as immunosuppressants in individuals undergoing treatment.

## Materials and methods

### Plasmids

The BID CDS was excised from a pTM1-2 vector encoding BID using NcoI and SpeI and ligated into NcoI- and XbaI-digested pGL3B-IL29/ISG56 promoter-luc reporters. Promoters and joined tBID CDS were then amplified with forward primers #1 (ISG56 promoter) or #2 (IL29 promoter) and reverse primer #3. PCR products were ligated to pLVX-tight-Neo (Clontech, Germany) using ClaI and SpeI restriction sites. lentiCRISPRv2 and GeCKOv2.0 human genome-wide sgRNA library were purchased from Addgene. The PTPRT gene was purchased from Kazusa DNA Research Institute (Japan) and amplified by primers #4–#5; the KDM2A and RBM39 genes were amplified from cDNA of PH5CH cells via primers #6–#7 and #8–#9, respectively. RBM39 siRNA-resistant mutants (primers #14–#15 and #16–#17) and IRF3 sgRNA-resistant mutants (primers #18–#19 and #20–#21) were generated by PCR. All sequences above were cloned into pWPI vector as previously described (21). The IRF3 promoter sequences described previously (65) were amplified from genomic DNA of PH5CH cells and cloned into the pGL3-basic vector via primers #22–#23. The primer sequences for cloning are listed in **Supplementary Table S5**.

### Cell culture

The PH5CH cells were a generous gift from K. Shimotohno. The Huh7.5 cells were a kind gift from Charles Rice. A549 cells were obtained from ATCC, while A549 IFNAR/LR KO cells (27), Huh7-Lunet cells (28), Huh7.5-RIG-I and Huh7.5-MDA5 (29), and HepG2-NTCP (30) have been described before. HepG2/C3A were obtained from ATCC. Primary human hepatocytes (PHH) were purchased from BioIVT and cultured in Williams’ E media containing 1% (v/v) Glutamax, 1% (v/v) non-essential amino acids, 1% (v/v) penicillin/streptomycin (all from Gibco, Germany), 0.2% (v/v) normocin (Invivogen, USA), 2% (v/v) B27 (Gibco, Germany), 1% (v/v) N2 supplement (Gibco, Germany), 100 mM nicotinamide (Sigma-Aldrich, USA), 1.25 mM N-acetylcysteine (Sigma-Aldrich, USA), 10 μM Y27632 (Peprotech, USA), and 1 μM A83-01 (Tocris, UK). The cells, unless specifically mentioned, were cultured in Dulbecco’s modified eagle medium (Life Technologies, Darmstadt, Germany) supplemented with 10% fetal bovine serum, nonessential amino acids (Life Technologies), 100 U/mL penicillin, and 100 ng/mL streptomycin (Life Technologies) and cultivated at 37°C and 5% CO_2_. PH5CH-KDM2A, PH5CH-RBM39.Esc, PH5CH-RBM39 G268V, PH5CH-IRF3, and A549-IRF3 cells were kept under selection pressure of 1 mg/mL G418 (Geneticin) (Life Technologies, USA), while Huh7-Lunet-TLR3, Huh7.5-RIG-I, and HepG2-NTCP cells were maintained under selective pressure of 1 μg/mL puromycin (Sigma-Aldrich, Germany). The Huh7.5-MDA5 cells were kept under antibiotic pressure of 5 μg/mL blasticidin (Sigma-Aldrich, Germany).

### siRNA transfection

The used siRNAs were purchased from Horizon Discovery, UK, unless specifically mentioned. For validation of the candidate genes from the CRISPR/Cas9 screen, ON-TARGETplus SMARTpools were used, covering each gene with a mixture of four siRNAs. siMAVS was purchased from Sigma Aldrich, Germany. For siRNA transfection in a six-well plate, cells with 60%–70% confluency were seeded 16 h prior to the experiment, and 1.5 μM siRNA was mixed with 9 μL Lipofectamine RNAiMax (Thermo Fisher Scientific, USA) in OptiMEM according to the manufacturer’s instructions. After 20 min of incubation at room temperature (RT), the mixture was added into the medium. The cells were incubated for 48 h and used for further experiments.

### Drug treatments

#### Poly(I:C) stimulation

Poly(I:C) (HMW) was used (Invivogen, USA) following the manufacturer’s instructions. To specifically trigger TLR3, 50 μg/mL poly(I:C) was added directly into the medium. For TLR3, RIG-I, and MDA5 stimulation, transfection was performed with 0.5 μg/mL poly(I:C) mixed with Lipofectamine2000 (Thermo Fisher Scientific, USA) in OptiMEM (Gibco, USA) according to the manufacturer’s instructions. After 20 min of incubation at RT, the mixture was added into the medium. The cells were stimulated for 6 or 8 h and then harvested for RNA or protein extraction.

#### Indisulam treatment

Indisulam was utilized (Sigma Aldrich, Germany) solved in DMSO at a final stock concentration of 10 mM. Indisulam or the same amount of DMSO as control was added to the cell medium at the indicated concentrations; the cells were incubated for the indicated time and treated further with either poly (I:C) or virus infection. At the indicated time points, the cells were harvested for RNA extraction or protein analysis.

#### IFN stimulations

IFNα2 and IFNλ1 were purchased from PBL, USA and Peprotech, USA, respectively. Added to the medium were 50 IU/mL IFN2α and 10 ng/mL IFNλ1. The cells were stimulated for 24 h and collected for RNA extraction.

### Primary mouse hepatocyte isolation

C57BL6/N wild-type mice (9 to 10 weeks, male) were housed in a specific-pathogen free (SPF) barrier at the Interfakultäre Biomedizinische Forschungseinrichtung (IBF) animal facility at Heidelberg University (Germany). The mice were maintained on a standard diet with *ad libitum* access to food and water under a constant light–dark cycle. The animal experiment was approved by the Regierungspräsidium Karlsruhe (T47/22). The mice were sacrificed; the abdomen was cleaned with ethanol and then opened to expose the inferior cava vein and the portal vein. The inferior cava vein was cannulated and washed with pre-warmed Liver Perfusion Medium (Thermo Fisher, USA) for about 3 to 4 minutes until the liver became blood free. Afterward, the liver was perfused with Liver Digest Medium (Thermo Fisher, USA) until it was digested. The liver without gallbladder was transferred to a Petri dish. Cold Hepatocyte Wash Medium (Thermo Fisher, USA) was added, and the organ liberating cells were de-capsuled. Subsequently, the cells were sequentially passed through 100- and 70-µm cell strainers into a 50-mL falcon tube and centrifuged at 50*g*, 4°C for 2 min, to separate liver non-parenchymal cells (NPC) that remained in the supernatant. Next, the hepatocyte (HC) pellet was washed with Hepatocyte Wash Medium (Thermo Fisher, USA) once and re-suspended in Williams’ E Medium, Glutamax Supplement (Thermo Fisher, USA) with 4% FBS and 1% pen-strep and seeded 5 × 10^4^ cells/cm^2^ in a collagen-coated plate (Collagen I Rat Protein, Tail, Thermo Fisher, USA).

### CRISPR/Cas9 knockout screening

For genome-wide CRISPR/Cas9 screen, PH5CH and Huh7-Lunet-TLR3 cells were transduced with lentiviral vectors containing the tBID death reporter and Cas9 (lentiCRISPRv2) (31). Neomycin and puromycin were added for selection. After that, PH5CH and Huh7-Lunet-TLR3 cells stably expressing the tBID death reporter and Cas9 were transduced with lentiviral vectors containing the GeCKOv2.0 human-genome-wide sgRNA library (31) (Addgene, USA) at MOI = 0.3. The GeCKOv2.0 human-genome-wide sgRNA library includes a total of 122,411 unique sgRNAs targeting 19,050 genes (with six sgRNAs per gene) and 1,864 miRNAs (with four sgRNAs per miRNA) and a total number of 1,000 different non-targeting sgRNAs as controls.

After 24 h, puromycin was added for selection. On day 7 post-transduction, 50 µg/mL poly(I:C) or PBS as control was added to the medium to exclusively activate the TLR3 pathway. The experiments were performed in two independent biological replicates with two technical replicates each. Surviving cells were collected at 72 h post-stimulation, and genomic DNA was extracted using the NucleoSpin Blood L kit (Macherey-Nagel, Germany) according to the manufacturer´s instructions. Amplicons were amplified, barcoded by PCR, and purified again via agarose gel separation using the NucleoSpin Gel and PCR Clean-up kit (Macherey-Nagel, Germany) before being pooled and submitted to GATC Biotech (Germany) for Illumina NGS (Illumina HiSeq, 50 bp single reads, 240 million reads total, 20% PhiX DNA).

The NGS data was firstly de-multiplexed to allocate the respective reads in the pooled population to each sample. Reads were counted for each gRNA in every sample and then normalized to the total number of reads in this sample as well as the complexity sample taken 1 day after the transduction of the lentiviral library, yielding normalized read counts.

The mean values of normalized read counts of the four technical replicates were generated for each of the four experiments. Based on mean values, fold changes were calculated between poly(I:C)- and PBS-treated samples. gRNAs with a higher fold change than a threshold were considered as primary hits. The threshold was adjusted for each cell line to yield about 2,000 top-ranked gRNAs for each experiment. Thresholds were 2.5× change in Huh-7-Lunet-TLR3 cells and 7× change in tPH5CH cells. Preliminary hits were furthermore filtered for occurrences of at least five upregulated gRNAs per gene in all datasets combined and the occurrence of at least one gRNA per gene in at least three independent datasets. This yielded 50 candidate genes and five candidate miRNAs.

### CRISPR/Cas9 knockout clones

To generate PH5CH IRF3 single knockout (KO) clones, PH5CH cells were transduced with the lentiCRISPRv2-IRF3 and lentiviral vectors encoding for Cas9 (31) (see “Plasmids”). After puromycin selection, the cells were seeded into 96-well plates for screening of stable single clones.

### Real-time quantitative PCR

Total RNA was isolated from cultured cells using the NucleoSpin RNA Plus kit (Macherey-Nagel, Germany). RT-qPCR was performed as described before (29). For gene expression analysis, cDNA was generated from RNA samples using the High-Capacity cDNA Reverse Transcription Kit (Thermo Fisher Scientific, USA). It was used for qPCR analysis with the 2x iTaq Universal SYBR Green Supermix (Bio-Rad, Germany). Reactions were performed on a CFX96 Touch Real-Time PCR Detection System (Bio-Rad, Germany) as follows: 95°C for 3 min, 95°C for 10 s, and 60°C for 30 s. mRNA fold change was calculated normalizing on glyceraldehyde-3-phosphate dehydrogenase (GAPDH) mRNA expression relative to untreated/uninfected/mock transfected cells. For IRF3 isoform mRNA measurements, cDNA was prepared using oligo(dT)18 primers (Thermo Scientific, Germany). mRNA expression fold change was normalized to GAPDH and hydroxymethylbilane synthase (HMBS) expression levels relative to siNT-transfected cells. Primers IRF3-228 + 203 were designed to target both IRF3-228 and IRF3-203 stretches; the other IRF3 primers were designed to target specific exons or introns of individual isoforms. IRF3-203 mRNA expression was calculated by subtracting the expression of IRF3-228 from that of IRF3-228 + IRF3-203. All qPCR primers are listed in **Supplementary Table S5**.

### Western blot

Cells were lysed in protein lysis buffer (50 mM Tris-HCl, pH 7.4, 150 mM NaCl, 1% Triton-X 100, protease inhibitor) on ice for 45 min. Cell lysates were then centrifuged at 14,000*g* for 15 min at 4°C to exclude cell debris. Supernatants were collected and mixed with 6x Lämmli buffer (0.375 M Tris-HCl, 9% SDS, 50% glycerol, 0.03% bromophenol blue, and 9% β-mercaptoethanol), boiled at 95°C for 5 min, and cooled down on ice for 2 min. Samples were separated by 10% SDS-PAGE and then blotted onto polyvinylidene difluoride (PVDF) membranes (Merk Millipore, Germany). Membranes were then blocked with 5% milk in PBST (0.05% Tween) or 5% BSA in TBST (0.01% Tween) and incubated with the indicated antibodies. Membranes were imaged via the ECL Plus Western Blotting Detection System (Pierce, GE Healthcare, Little Chalfont, UK) according to the instructions of the manufacturer. Signal was recorded using the Advance ECL Chemocam Imager (Intas Science Imaging, Germany). The quantification of western blots was performed by Fiji.

### Lentivirus preparation and transduction

There were 5 × 10^6^ HEK293T cells seeded in 10-cm dishes 16 h before transfection. On the next day, the target plasmid (5.14 µg) together with pCMV-Gag-Pol (5.14 µg) and pMD2-VSVG (1.71 µg) was mixed in 400 µL OptiMEM (Gibco, USA). Moreover, 364 µL of OptiMEM was separately mixed with 36 µL polyethylenimine (PEI). The two mixtures were combined and incubated for 20 min at RT and then added to the cells as previously described (32). After 2 days, lentivirus was collected, filtered using a 0.45-µm filter (Sigma Aldrich, Germany) to exclude cell debris, and stored at -80°C or used for transduction. PH5CH-IRF3 and A549-IRF3 stable cells were produced by the transduction of lentiviral particles encoding for IRF3 under control of the Rosa26 promoter (IRF3 sgRNA-resistant mutant) as previously described (29) in the abovementioned PH5CH IRF3 KO cells. PH5CH-KDM2A, PH5CH-RBM39.Esc, and PH5CH-RBM39 G268V were generated by the lentiviral transduction of particles containing corresponding plasmids.

### Cell viability assay

Cell viability upon transduction of tBID death reporter was assessed in 96-well plates through the WST-1 assay (Roche, Switzerland) according to the manufacturer’s instructions. The cell viability of indisulam-treated cells was accessed by CellTiter-Glo^®^ 2.0 Assay (Promega, Germany) according to the manufacturer’s instructions.

### ELISA

Supernatants were collected from stimulated cells, and IFNβ protein levels were measured by LumiKineTMXpress hIFN-β 2.0 (InvivoGen, Germany) according to the manufacturer’s instructions.

### Luciferase reporter assay

pGL3-982 or pGL3-149 Firefly reporter, together with pRL-CMV Gaussia (Promega, USA) reporter as reference, was transfected into PH5CH RBM39 knockdown cells using Lipofectamine2000 (Thermo Fisher Scientific, USA) according to the manufacturer’s instructions. After 24 h, the cells were lysed by the addition of Luciferase lysis buffer (1% Triton X-100, 25 mM glycylglycine, 15 mM MgSO_4_, 4 mM EGTA, 10% glycerol, pH 7.8, and 10 mM DTT), and luciferase activity (Relative Ligh Units, RLU) was measured by a Mitras LB940 luminometer (Berthold Technologies, Germany). RVFV-RLuc assay was performed as described before (33). At 24 h after infection, the infected cells were washed once with PBS and immediately lysed with luciferase lysis buffer. Cell lysates were stored frozen at -80°C to ensure proper lysis, and the Renilla luciferase signal was measured by injecting 300 µL of luciferase assay buffer (15 mM K_3_PO_4_ pH 7.8, 25 mM glycylglycine, pH 7.8, 15 mM MgSO_4_, and 4 mM EGTA) supplemented with coelenterazine (102173, PJK, Germany), followed by read-out of the signal with a 480-nm high-sense filter (480m20BREThs, Berthold) using the Mitras2 multimode plate reader (LB942, Berthold).

### RNA sequencing

RNA sequencing and RNA quality control were performed by the Genomics and Proteomics Core Facility of the German Cancer Research Centre using the Illumina Hiseq NovaSeq 6000 with RNA seq reads in 2 × 100-bp format. For sample preparation, 1.5 × 10^5^ cells were seeded in a six-well plate. The cells were either transfected with siRBM39 or siNT as control or treated with 1 µM indisulam or the same amount of DMSO as control. At 48 h after transfection, the cells were lysed, and total RNA was extracted via NucleoSpin RNA Plus kit (Macherey-Nagel, Germany) following the manufacturer’s instructions. RNA was quantified using OD measurement at 260 nm.

Human reference transcriptome (Human Gencode v40) mapping and quantification of the transcripts were performed using the salmon package with GC bias correction (34). Differential expression analysis was performed using DESeq2 (35), DTU analysis was performed with DRIMseq (36), and gene set enrichment analysis was performed with clusterProfiler (37) in R.

### Virus infection

For Sendai virus (SeV) infection, 6 × 10^4^ A549 cells were transfected with siRNA targeting RBM39 or treated with 1 µM indisulam/DMSO. After 48 h, the cells were infected with SeV (MOI = 1) in PBS with 0.3% BSA at RT. At 1 h after infection, the cells were washed with PBS, and complete medium was added. Cells were collected after 24 h for further analysis.

For vesicular stomatitis virus (VSV) infection, 6 × 10^4^ A549 cells were treated with 1 µM indisulam or DMSO control for 48 h. Afterward, the cells were infected with recombinant VSV-G (rVSV-G) (MOI = 0.1) for 24 h. Cells were collected for further analysis.

For Rift Valley fever virus (RVFV) infection, 2 × 10^4^ A549 cells were seeded into 24-well plates. On the following day, the cells were treated with 0.1 µM indisulam or DMSO for 48 h and then inoculated with RVFV-RLuc (33) at MOI of 0.01 in DMEM with 2% FCS. After 24 h of infection, the cells were washed once with PBS, lysed in 100 µL of luciferase lysis buffer, and frozen at -80°C for further analysis.

For hepatitis E virus (HEV) (Genotype 3 Kernow C1 P6, accession number: JQ679013, a kind gift from Suzanne Emerson, NIH) (38) and hepatitis D virus (HDV) infection, 2.5 × 10^4^ HepG2/C3A cells and HepG2-NTCP cells, respectively, were seeded in collagen (Corning, USA)-coated plates and treated with 0.1 µM indisulam or the same amount of DMSO for 48 h. Subsequently, the cells were infected with HEV (MOI = 4) in MEM with 10% FBS or HDV (MOI = 4) in DMEM with 1.5% DMSO and 4% PEG. At 24 h after infection, the cells were washed with PBS. The cells were incubated for 5 days and collected for further analysis.

### Mass spectrometry

PH5CH cells were treated with siRBM39 or indisulam for 48 h, and siNT and DMSO were used as control. Afterward, cells were harvested, lysed in 300 µL sodium dodecyl sulfate (SDS) lysis buffer (4% SDS, 10 mM DTT, 55 mM iodoacetamide (IAA), and 50 mM Tris–HCl, pH 7.5), and heat-inactivated (95°C for 10 min). The samples were sonicated (4°C, 15 × 30 s on and 30 s off setting), and the protein lysate was precipitated using pre-chilled acetone (−20°C) at a final concentration of 80% acetone (v/v) overnight. Precipitates were pelleted and washed two times with pre-chilled 80% acetone (-20°C). Pellets were air-dried at room temperature and resolubilized by adding 40 µL thiourea buffer (6 M urea and 2 M thiourea (U/T) in 10 mM HEPES, pH 8.0). Protein concentration was determined by BCA assay (Pierce) according to the manufacturer’s instructions and adjusted to 50 µg with thiourea buffer. Lysates were digested overnight (25°C, 16 h, 1,200 rpm) using LysC (1:50 w/w, enzyme/protein, Wako) and trypsin (1:50 w/w, enzyme/protein, Promega) per replicate. The samples were prepared for C18-stage tipping by adding 1/5 of loading buffer [10% acetonitrile (ACN) and 3% trifluoroacetic acid (TFA)] to inactivate proteases (TFA) and enhance the solubility (ACN) of samples. Peptides were desalted with three-layer C18 stage tips (3M). Columns were equilibrated with 200 µL methanol (RT, centrifuged at 300*g*, 4 min), washed twice with 200 µL washing buffer [0.5% acetic acid (AA)], loaded with the sample, washed twice with 200 µL washing buffer (0.5% AA), and eluted (80% acetonitrile, 0.5% AA). All steps were executed with 500*g* at RT. The eluate was evaporated (45°C, vacuum) and resuspended in MS buffer (2% acetonitrile, 0.3% TFA). Peptide concentrations were measured at 280 nm (Nanodrop 2000, Thermo Scientific) and subsequently equalized to 0.25 µg/µL. The peptides were loaded onto a 2-cm trapping column (75 µm diameter; ReproSil-Pur C18-AQ 5 µm resin; Dr. Maisch) at 5 µL/min 0.1% formic acid (FA) in water using the UltiMate 3000 RSLCnano System (Thermo Fisher). The peptides were eluted onto a 42-cm analytical column (75 µm diameter; ReproSil-Pur C18-AQ 1.9 µm resin; Dr. Maisch) over a 120-min gradient at a flow rate of 300 nL/min and the column oven operating at 50°C using solvent B (0.1% FA and 5% DMSO in acetonitrile), starting from 4% to 32% over 110 min, then to 80% over 2 min, and kept at 80% for another 2 min, to 2% over 2 min, and kept at 2% for 3 min. nanoLC solvent A was 0.1% FA and 5% DMSO in HPLC-grade water.

Spectra were acquired with an Orbitrap Eclipse Tribrid mass spectrometer (Thermo Fisher Scientific) running Tune 3.5 and Xcalibur 4.5 and equipped with a nano-electrospray source (Thermo Fisher Scientific). Spray voltage was set to 2.1 kV, RF lens at 40%, and heated capillary at 275 °C. The Eclipse mass spectrometer was operated in data-independent acquisition (DIA) and positive ionization mode. MS1 full scans (360–1,300 m/z) were recorded at a resolution of 120,000 (Orbitrap) using a normalized automatic gain control (AGC) target value of 100% and maximum injection time of 50 ms. Peptides were fragmented using higher energy collision-induced dissociation (HCD) collision energy set to 30%. MS2 scans (200–1,800 m/z) were acquired over a total of 40 DIA segments with variable isolation windows and with a window overlap of 1 m/z. The scan resolution in the Orbitrap was set to 30,000 with a normalized AGC target value of 1000% and a maximum injection time of 54 ms.

Raw MS data files of the experiments conducted in data-independent acquisition (DIA) mode were processed with DIA-NN version 1.8.1 (39). An *in silico*-predicted spectral library composed of the human proteome (UniprotKB reference proteome, UP000005640, downloaded October 2021) and common contaminants (MaxQuant contaminants.fasta) with trypsin as digestion enzyme and one missed cleavage specified was generated in DIA-NN. Subsequently, the acquired raw files were processed in library-free mode using DIA-NN default settings, and the match between runs function was enabled. The R package iq was used to generate MaxLFQ protein abundance output with a Lib.Q.Value and Lib.PG.Q.Value filter setting of 0.01 (40). The ProteinGroups output file was processed with Perseus (version 1.6.15.0). The intensities were expressed as Log2(x) and imputed (replacing missing values from normal distribution, with 0.3; downshift 1.8), and annotation was included. Statistical significance evaluated by “two-sample tests” (Student’s *t*-test with permutation-based FDR 0.05 and 250 number of randomizations).

### Statistical analysis

Independent biological replicates are denoted with *n*-numbers. To test for significance, two-tailed unpaired Welch’s test was performed using GraphPad Prism 8 software (GraphPad Software, La Jolla, CA, USA) unless specifically mentioned (∗*p* < 0.05; ∗∗*p* < 0.01; ∗∗∗*p* < 0.001; ∗∗∗∗*p* < 0.0001). For proteomics, statistical significance was evaluated by “two-sample tests” (Student’s *t*-test with permutation-based FDR 0.05 and 250 number of randomizations). For the analysis of RNAseq data, *p*-values were calculated with the Wald test and corrected for multiple testing according to Benjamini–Hochberg.

## Results

### A genome-wide CRISPR/Cas9 Screen identifies novel factors contributing to TLR3 signaling

The initial aim of this study was the identification of novel factors contributing to the regulation of cell intrinsic innate immune responses, in particular focusing on TLR3-mediated response in hepatocytes.

**Figure 1:**
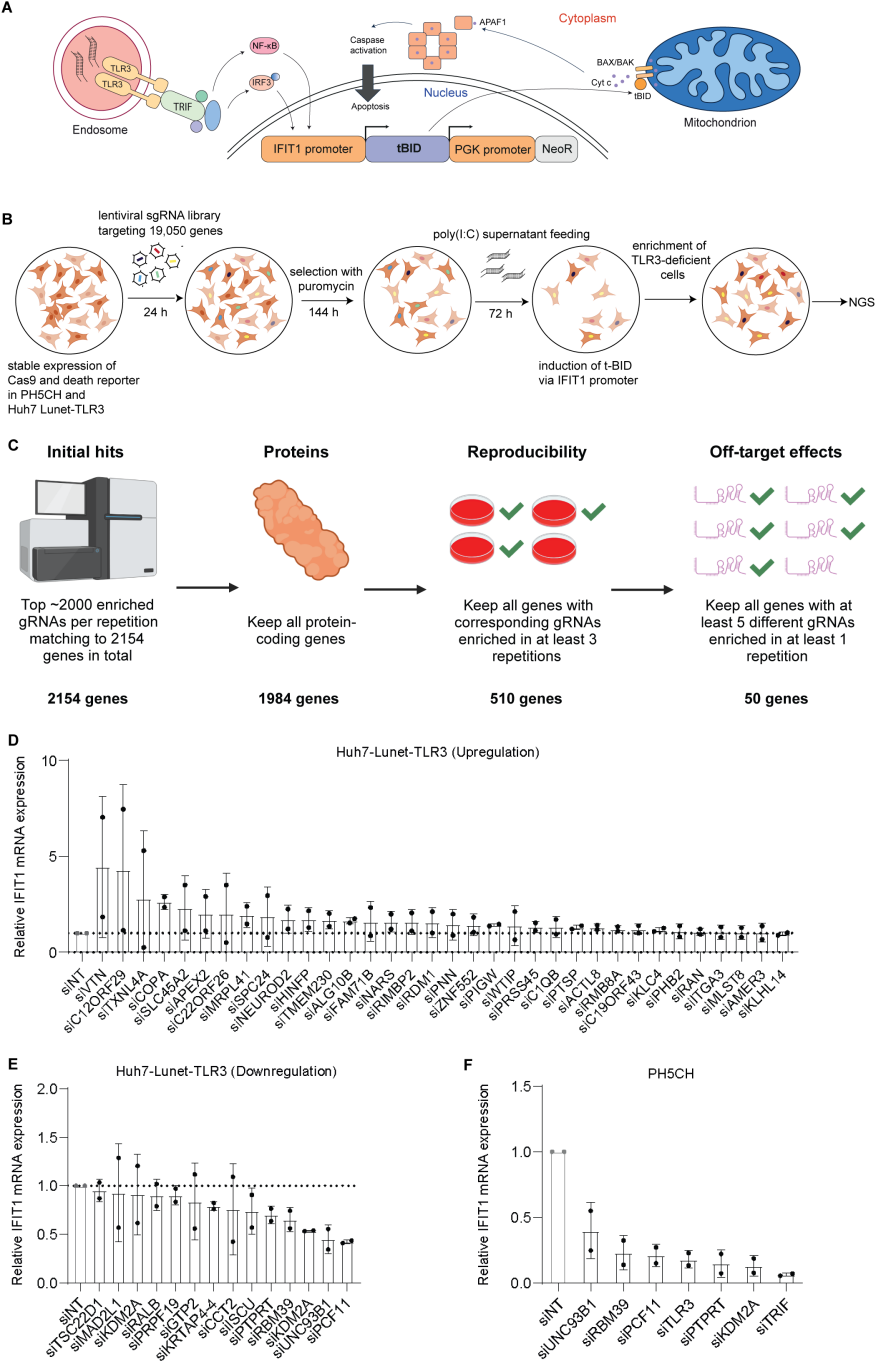
A genome-wide CRISPR/Cas9 screen identifies potential TLR3-related factors. **(A)** Schematic of the lentiviral truncated BID (tBID) death reporter system. The tBID expression is driven by the *IFIT1* promoter, while the expression of the neomycin selection marker (NeoR) is under the control of the constitutive *PGK* promoter. Working principle: (1) dsRNA is taken up via endocytosis and transported to the endosome. (2) The endosomal TLR3 recognizes dsRNA and triggers signaling via TRIF, resulting in the activation of transcription factors IRF3 and NF-κB. (3) Activated transcription factors induce the expression of tBID by binding the *IFIT1* promoter. (4) tBID associates with Bcl-2 proteins BAX and BAK to form a complex that permeabilizes the outer mitochondrial membrane and mediates the release of cytochrome c (Cyt c) into the cytoplasm. (5) Cyt c binds APAF1, triggering its oligomerization and binding to Pro-Caspase-9. Activated Caspase-9, in turn, activates Caspase-3 and -7, inducing apoptosis. **(B)** Workflow of the CRISPR/Cas9 screen. PH5CH and Huh7-Lunet-TLR3 stably expressing the death reporter and Cas9 were transduced with the genome-wide lentiviral CRISPR sgRNA library and selected with puromycin for stable expression. TLR3 stimulation with poly(I:C) induced the expression of tBID and thus apoptosis in case of intact TLR3 signaling. Then, surviving TLR3-deficient cells were enriched and collected for next-generation sequencing (NGS). **(C)** Illustration of the filtering steps to generate the final hit list. For each experiment independently, the ~2,000 most enriched gRNAs were determined in the NGS dataset and matched to their respective target genes, creating a list of 2,154 initial genes of interest. Subsequently, it was only focused on protein-coding genes. To ensure reproducibility, only genes with enriched gRNAs in three out of four repetitions were considered; this also eliminated genes showing enrichment in only one of the cell lines used. Lastly, genes were filtered to have at least five out of six available gRNAs enriched in at least one repetition to remove hits driven by potential off-target effects of just a subset of gRNAs. **(D–F)** A total of 50 candidate genes were selected and subjected to siRNA silencing for 48 h; then, the cells were stimulated with 50 μg/mL poly(I:C) supernatant feeding for 8 h. *IFIT1* mRNA was measured by RT-qPCR normalized to GAPDH and expressed as relative expression to siRNA non-targeting (siNT)-treated samples. Knockdown of the candidates upregulated **(D)** or downregulated **(E)***IFIT1* mRNA expression in Huh7-Lunet-TLR3 cells. Knockdown of *RBM39*, *PCF11*, *PTPRT*, and *KDM2A* in PH5CH cells reduced *IFIT1* mRNA expression in PH5CH cells **(F)**. siUNC93B1, siTLR3, and siTRIF were used as positive controls. The data are from three biological replicates (*n* = 3); error bars indicate standard deviation (SD).

To establish a positive selection system, we developed a death reporter system expressing the suicide gene truncated BH3 interacting domain death agonist (tBID) under the transcriptional control of the interferon-induced protein with tetratricopeptide repeats 1 (IFIT1) promoter (**Figure 1A**). We chose this promoter since it is activated and also independent of IFN stimulation by IRF3 and shows a high level of induction after stimulation. Upon activation of the TLR3 pathway, tBID expression is induced, resulting in apoptosis (41). For the screening, we chose PH5CH (42), a non-neoplastic, immortalized hepatocyte cell line expressing all PRRs responsive to dsRNA, and Huh7-Lunet-TLR3 (29), a hepatocellular carcinoma (HCC)-derived cell line requiring ectopic expression of PRRs for efficient response to dsRNA. The tBID reporter was transduced into both cell lines for stable expression. Although IFIT1, like most ISGs (43), is expressed at detectable levels in the absence of stimulation, no obvious cell death due to basal tBID expression was observed upon selection of the stable cell lines. To evaluate the approach, TLR3-specific stimulation was achieved by the addition of the synthetic dsRNA analog polyinosinic:polycytidylic acid (poly(I:C)) to the cell culture supernatant. Supernatant delivery of poly(I:C) in the absence of transfection reagents has been shown to exclusively mediate TLR3 activation in both models (32, 44, 45). Indeed the supernatant feeding of poly(I:C) resulted in efficient tBID-associated, TLR3-dependent cell death only upon activation of the *IFIT1* promoter in both cell lines, in contrast to the empty vector (**Supplementary Figure S1A**). Subsequently, we transduced the IFIT1-tBID cell lines with lentiviral vectors encoding Cas9 along with the GeCKOv2.0 human genome-wide single guide RNA (sgRNA) library (31) that includes a total of 122,411 unique sgRNAs targeting 19,050 genes and 1,864 miRNAs (**Figure 1B**). After selection with puromycin, we added 50 µg/mL poly(I:C) to the medium to activate the TLR3 pathway or PBS as a control. As a consequence, cells with an intact TLR3 pathway were killed due to the induction of tBID expression, whereas cells expressing guide RNAs targeting genes involved in TLR3 signaling survived, resulting in the enrichment of such sgRNAs. We then analyzed the sgRNA population of stimulated and non-stimulated cells at 72 h post-stimulation by next-generation sequencing (NGS) to identify target genes interfering with the TLR3 pathway.

Candidate genes were determined by being enriched beyond a cell-line-specific threshold calibrated to yield about 2,000 overrepresented sgRNAs for each cell line. Due to the expected background of the screen, we needed to narrow down the enriched genes to obtain primary screen hits. We filtered primary candidate genes using a thresholding strategy that was calibrated to include three positive controls [TRIF, TLR3, and unc-93 homolog B1 (UNC93B1)] for each cell line (46–50). The chaperone UNC93B1 was chosen due to its essential role in TLR3 trafficking to the endosome, rendering it essential for functional TLR3 responses (48–50). Still it is not a component of the signaling pathway, thereby reflecting alternative modes of action of potential candidate hits. A gene was considered as a primary hit in case five out of six different sgRNAs were found enriched in the poly(I:C)-treated samples of at least one replicate and the presence of at least one sgRNA targeting a gene in two out of three replicates. After such filtering, 50 protein-coding genes were chosen for functional validation, whereas five microRNAs were excluded despite matching our criteria (**Supplementary Table S1**; **Figure 1C**). The protein-coding genes were validated by siRNA knockdown in Huh7-Lunet-TLR3 cells (**Figures 1D, E**). However, the knockdown of most candidates resulted in unimpaired or even upregulated TLR3 responses (**Figure 1D**). The high number of false positive candidate genes was not fully unexpected since approximately 25% of the cells survived poly(I:C) stimulation despite the presence of the IFIT1-tBID reporter. Since this pool of non-responsive cells would not need a specific gRNA for rescue, it was supposed to generate a high background of surviving cells with arbitrary sgRNAs (**Supplementary Figure S1A**), resulting in non-specific enrichment. Only the knockdown of a few genes reduced the TLR3 response to levels comparable to the positive control (siUNC93B1). The four candidates with the strongest phenotype—RBM39, lysine demethylase 2A (KDM2A), protein tyrosine phosphatase receptor type T (PTPRT), and cleavage and polyadenylation factor II subunit (PCF11)—were chosen for further validation in PH5CH cells.

Indeed the knockdown of these factors strongly attenuated innate immune responses in PH5CH cells as well (**Figure 1F**). However, PCF11 was not followed up due to its general role in termination of transcription (51). *PTPRT* mRNA turned out not to be detectable in Huh7 or PH5CH cells, and the overexpression of siKDM2A-resistant mutant in PH5CH cells failed to rescue the KDM2A phenotype (**Supplementary Figure S1B**), arguing for off-target effects of these siRNAs. Therefore, RBM39 was chosen for further studies.

### RBM39 knockdown reduces IRF3- but not NF-κB-induced genes upon TLR3 activation

**Figure 2:**
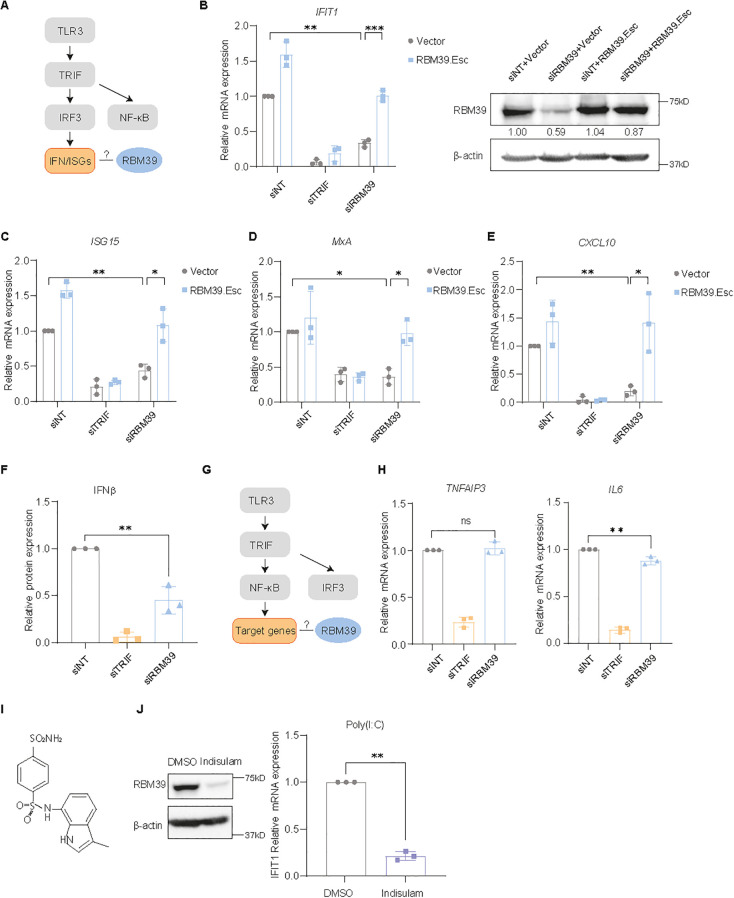
RBM39 is crucial for the TLR3 pathway. **(A)** Simplified schematic of the TLR3-IRF3 pathway. **(B–E)** RBM39 rescue experiment. PH5CH cells expressing RBM39.Esc or empty vector were transfected with siRBM39 or siNT/siTRIF as controls for 48 h and then supernatant-fed with 50 µg/mL poly(I:C) for 6 h. *IFIT1***(B)**, *ISG15***(C)**, *MxA***(D)**, and *CXCL10***(E)** mRNA was measured by RT-qPCR; RBM39 protein expression was measured by immunoblotting **(B)**. **(F–H)** PH5CH cells were transfected with siRNA for 48 h and then fed with 50 μg/mL poly(I:C) in the supernatant for 6 h. Secreted IFN-β protein **(F)** was measured by ELISA. Simplified schematic of the TLR3-NF-κB pathway **(G)**. *TNFAIP3***(H)** and *IL6***(H)** mRNA was quantified by RT-qPCR. **(I)** Chemical structure of indisulam. **(J)** PH5CH cells were treated with 1 µM indisulam or DMSO as control for 48 h and further stimulated with 50 µg/mL poly(I:C) in the supernatant for 6 h. RBM39 degradation was measured by western blot (left). *IFIT1* mRNA expression was measured by RT-qPCR (right). Relative expression to siNT control was shown; mRNA fold change was normalized to *GAPDH*. Data are obtained from three biological replicates (*n* = 3); error bars refer to SD (all panels). Statistical significance was assessed through Welch’s unpaired *t*-test. ns = not significant, * = p < 0.05, ** = p < 0.01, *** = p < 0.001.

Since PH5CH was considered the most authentic among the available hepatocyte models due to its non-neoplastic nature, we decided to continue here with an in-depth analysis of the specific role of RBM39 on TLR3-IRF3 signaling (**Figure 2A**).

First of all, an RBM39 siRNA-resistant mutant (RBM39.Esc) was able to rescue the *IFIT1* mRNA expression upon siRNA knockdown in both PH5CH (**Figure 2B**) and Huh7-Lunet-TLR3 cells (**Supplementary Figure S2**), demonstrating that the phenotype indeed was mediated by *RBM39* knockdown. To rule out that RBM39 might have an exclusive impact on *IFIT1* expression, which so far served as the sole readout in our validation, we further analyzed the modulation of other ISGs upon *RBM39* knockdown and TLR3 stimulation, including interferon-stimulated protein, 15 kDa (ISG15), MX dynamin like GTPase 1 (MxA), and C-X-C motif chemokine ligand 10 (CXCL10) in PH5CH cells. All tested ISGs showed a reduction in *RBM39*-silenced cells compared to the control, and RBM39.Esc overexpression again rescued the knockdown effect (**Figures 2C–E**), excluding the off-target effect of siRBM39 and suggesting that RBM39 has a broad impact on various ISGs. To confirm the impact of RBM39 on IRF3-mediated IFN induction on protein level, we additionally detected IFN-β secretion via ELISA upon *RBM39* knockdown and observed a significant decline here as well (**Figure 2F**).

Considering that the activation of PRRs not only leads to the production of various ISGs and IFN-β but also triggers NF-κB signaling, resulting in the production of cytokines and chemokines, we investigated a potential involvement of RBM39 in the NF-κB pathway (**Figure 2G**). However, the expression of two NF-κB-induced genes, tumor necrosis factor alpha-induced protein 3 (TNFAIP3) and interleukin 6 (IL6), was not or only mildly affected in PH5CH cells upon poly(I:C) activation and *RBM39* knockdown (**Figure 2H**), indicating a minor effect of RBM39 on the NF-κB pathway in this experimental setting.

Indisulam is a sulfonamide drug mediating the interaction between the E3 ligase DCAF15 and RBM39, leading to the ubiquitination and proteasomal degradation of RBM39 (**Figure 2I**) (23, 52, 53). Indeed indisulam dramatically reduced RBM39 expression in PH5CH, resulting in significantly attenuated *IFIT1* induction upon TLR3 activation (**Figure 2J**), further emphasizing the role of RBM39 on the TLR3 pathway.

Overall, our results suggest that RBM39 was involved in the direct induction of IFN-β and ISGs upon stimulation of TLR3 with extracellular dsRNA but had a minor influence on NF-κB signaling, suggesting that the IRF3 pathway was mainly affected.

### RBM39 controls the RIG-I/MDA5 pathway

**Figure 3:**
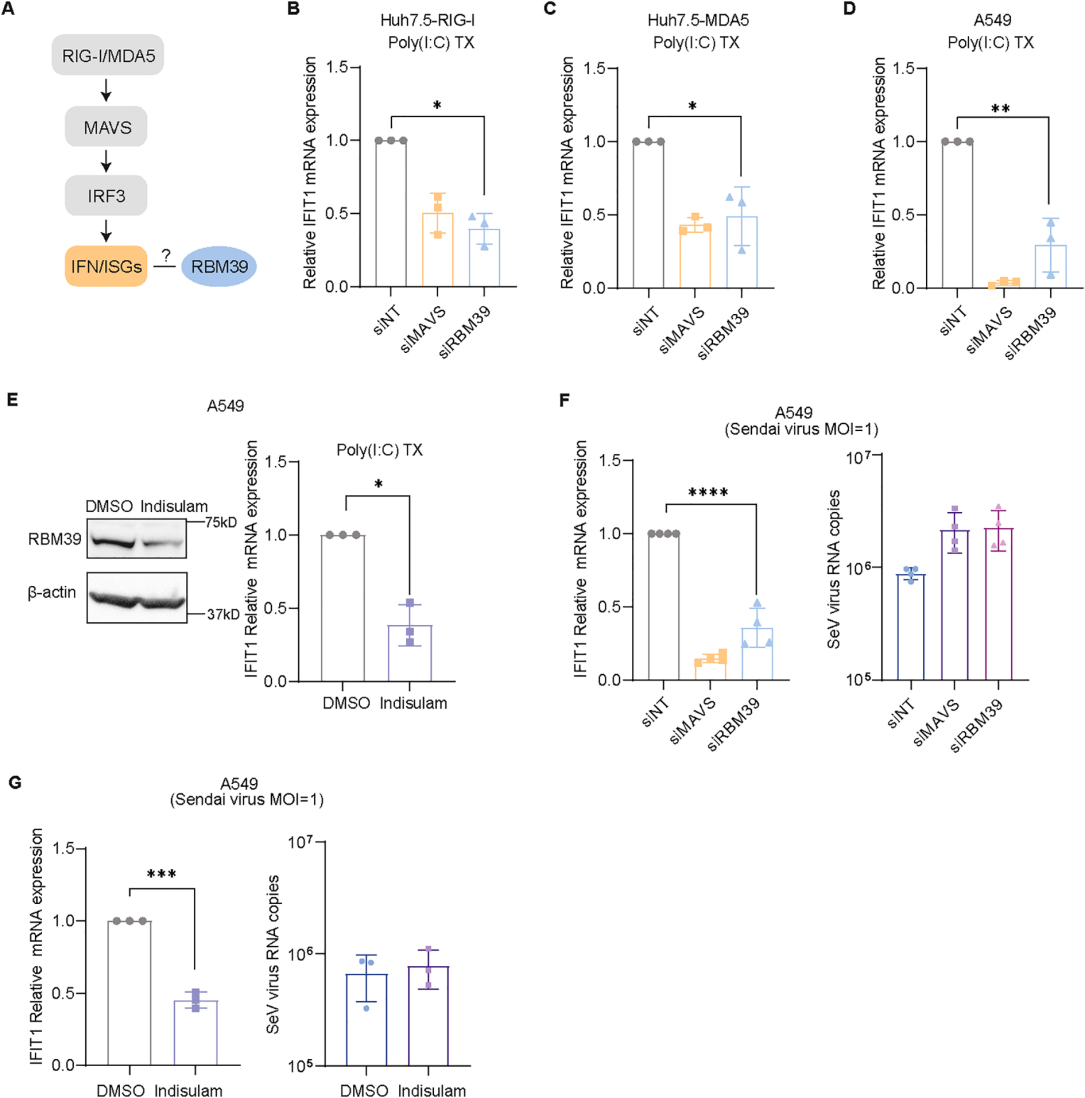
RBM39 participates in the RIG-I/MDA5 response. **(A)** Simplified schematic of the RIG-I/MDA5-IRF3 pathway. **(B–D)** Huh7.5 cells with ectopic RIG-I **(B)** or MDA5 **(C)** expression and A549 cells **(D)** were transfected with siRBM39 or siNT/siMAVS as control for 48 h and then transfected with 0.5 µg/mL poly(I:C) for 6 h*. IFIT1* mRNA was measured by RT-qPCR and is shown relative to siNT. **(E)** A549 cells were treated with indisulam or DMSO as control for 48 h and then transfected with 0.5 µg/mL poly(I:C) for 6 h. RBM39 degradation was determined by immunoblotting (left). *IFIT1* mRNA was quantified by RT-qPCR (right). **(F, G)** A549 cells were transfected with siRBM39 or siNT/siMAVS as control **(F)** or treated with 1 µM indisulam or DMSO as control **(G)**. At 48 h after treatment, the cells were infected with Sendai virus (MOI = 1) for 24 h. *IFIT1* mRNA (left) and Sendai virus RNA copies (right) were measured through RT-qPCR. mRNA fold change was normalized to *GAPDH*. The data were obtained from three biological replicates (*n* = 3); error bars refer to SD. Statistical significance was assessed through Welch’s unpaired *t*-test. * = p < 0.05, ** = p < 0.01, *** = p < 0.001, **** = p < 0.0001.

Given that Huh7.5 cells, a subclone of Huh7 (54), did not express functional PRRs, the ectopic expression of RIG-I and MDA5 allowed us to identify the function of RBM39 in other key innate immune pathways governed by IRF3 (**Figure 3A**) (29). Notably, silencing of *RBM39* decreased RIG-I- and MDA5-mediated *IFIT1* mRNA expression upon transfection of poly(I:C) (**Figures 3B, C**). To validate our finding in non-hepatic cell lines, we tested A549 cells, which are based on adenocarcinoma cells from human alveolar basal epithelium. These cells endogenously express RIG-I and MDA5, but not TLR3, and are regarded as innate immune-competent (55). Indeed the knockdown of *RBM39* in A549 cells reduced *IFIT1* induction in response to poly(I:C) similarly as observed for PH5CH and Huh7-Lunet (**Figure 3D**). Additionally, indisulam-induced RBM39 degradation also significantly reduced RIG-I/MDA5 responses in A549 cells (**Figure 3E**), collectively demonstrating that RBM39’s involvement in innate immunity is limited neither to hepatocytes nor to TLR3 signaling.

So far, our study exclusively relied on the stimulation of cell intrinsic innate immune responses using the synthetic dsRNA analog poly(I:C). To investigate the function of RBM39 in innate immunity in more physiological settings, we performed virus infection experiments with various viruses known to induce strong innate immune responses mediated by IRF3.

Sendai virus (SeV) is a model virus sensed by RIG-I (56). The knockdown of *RBM39* in A549 cells indeed resulted in a strong reduction of *IFIT1* mRNA expression, comparable to the one upon *MAVS* knockdown. We further assessed viral RNA abundance to ensure that RMB39 depletion would not inhibit viral replication, resulting in lower levels of viral stimuli. To this end, we found a slight, comparable increase for both conditions (**Figure 3F**). Indisulam was used as an alternative way to delete RBM39 in A549 cells consistently. It also attenuated SeV-induced *IFIT1* expression, again without affecting viral RNA loads (**Figure 3G**). Similar results were obtained for the infection of A549 cells with RVFV and vesicular stomatitis virus (VSV), HepG2/C3A cells with hepatitis E virus (HEV), and HepG2-NTCP cells with hepatitis D virus (HDV) upon indisulam treatment, resulting in reduced *IFIT1* mRNA induction, in the absence of inhibitory effects on viral RNA replication (**Supplementary Figure S3**).

Our data altogether demonstrate that RBM39 participated in the regulation of RIG-I and MDA5 pathways and substantially contributed to the cell intrinsic innate immune response to viral infections.

### RBM39 regulates basal IRF3 expression to modulate innate immune responses

**Figure 4:**
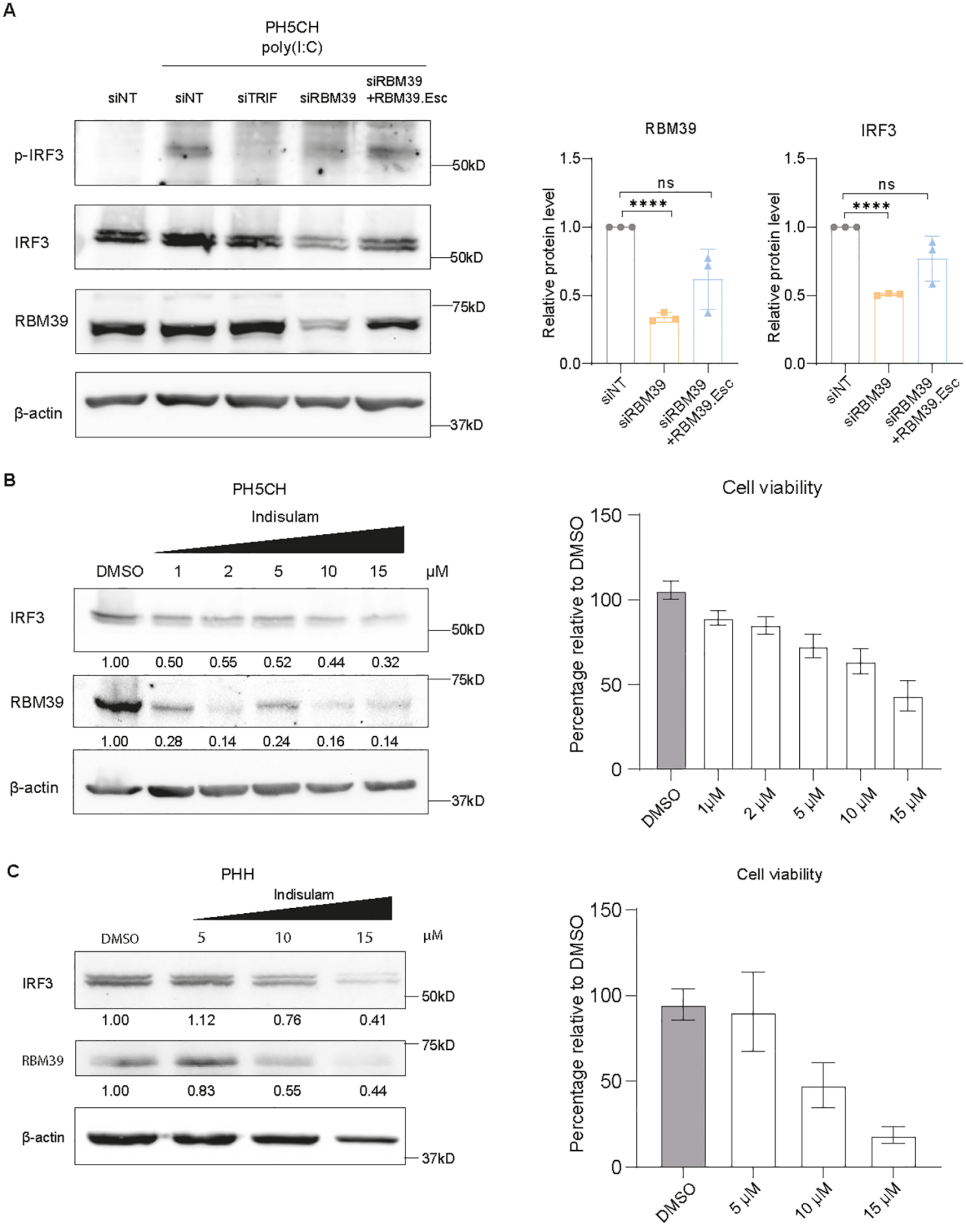
RBM39 controls the basal expression level of IRF3. **(A)** PH5CH cells or PH5CH RBM39.Esc cells were transfected with siRBM39 or siNT/siTRIF as controls for 48 h and then fed with 50 µg/mL poly(I:C) for 6 h. Phosphorylated IRF3 (p-IRF3), IRF3, RBM39, and β-actin protein expression levels were assessed by western blot (left). Quantification of three biological replicates is shown (right). **(B, C)** PH5CH cells **(B)** and primary human hepatocytes (PHH) **(C)** were treated with increasing concentrations of indisulam as indicated. IRF3, RBM39, and β-actin protein expression levels were detected through immunoblotting (left). Cell viability was measured via CellTiter-Glo luminescent cell viability assay (right) and normalized to DMSO control. Quantification of three independent experiments was expressed as average and is shown under each band. Protein expression was normalized to β-actin; relative protein levels to siNT or DMSO control are shown. The data shown are from three biological replicates (*n* = 3); error bars indicate SD. Statistical significance was assessed through Welch’s unpaired *t*-test. ns = not significant, **** = p < 0.0001.

In order to elucidate the mechanism involved in RBM39-mediated regulation of IFN and ISG induction, we firstly examined the phosphorylation of IRF3, a key event mediating IFN and ISG induction upon PRR activation by dsRNA. As expected, phosphorylated-IRF3 (p-IRF3) was undetectable in non-stimulated cells and strongly enhanced upon poly(I:C) stimulation. Intriguingly, *RBM39* knockdown resulted not only in a remarkable decrease in p-IRF3 but also in a significant reduction of total IRF3 protein levels, suggesting a regulation of the constitutive expression of IRF3 (**Figure 4A**).

To further validate the impact of RBM39 on IRF3 expression, we treated PH5CH cells with indisulam. Indeed the RBM39 protein levels were reduced in indisulam-treated cells in a dose-dependent manner, concomitant with lower IRF3 abundance and some cytopathic effect at higher concentrations (**Figure 4B**). In order to verify whether IRF3 depletion upon indisulam treatment was mediated by RBM39, we made use of an RBM39 mutant (G268V) reported to confer partial resistance to indisulam (23). Overexpression of RBM39 G268V partially rescued RBM39 indisulam-mediated degradation and thus restored IRF3 expression compared to cells expressing wild-type RBM39 (**Supplementary Figure S4A**). These results confirmed that indisulam affected IRF3 expression by targeting RBM39.

Similar results were obtained in A549 cells (**Supplementary Figure S4B**) and in primary human hepatocytes (PHH) (**Figure 4C**, left), with the latter indicating that the RBM39-mediated IRF3 depletion also appeared in primary cells, albeit requiring higher concentrations associated with substantial cytotoxicity (**Figure 4C**, right). In contrast, RBM39 was refractory to indisulam treatment in primary mouse hepatocytes (PMH) (**Supplementary Figure S4C**, left), probably due to the lack of expression of murine DCAF15 or resistance to indisulam (57), in line with the lack of cytotoxicity (**Supplementary Figure S4C**, right).

Above all, our results demonstrated that RBM39 governs the basal expression of IRF3, thereby exerting a crucial role in the activation of innate immune responses.

### Global transcriptome, splicing, and proteome analysis highlight the role of RBM39 in innate immunity

We next aimed for a comprehensive proteomic and transcriptomic analysis to identify other components of innate immunity affected by RBM39, comparing the impact of siRNA-mediated *RBM39* knockdown and indisulam treatment in PH5CH cells.

**Figure 5:**
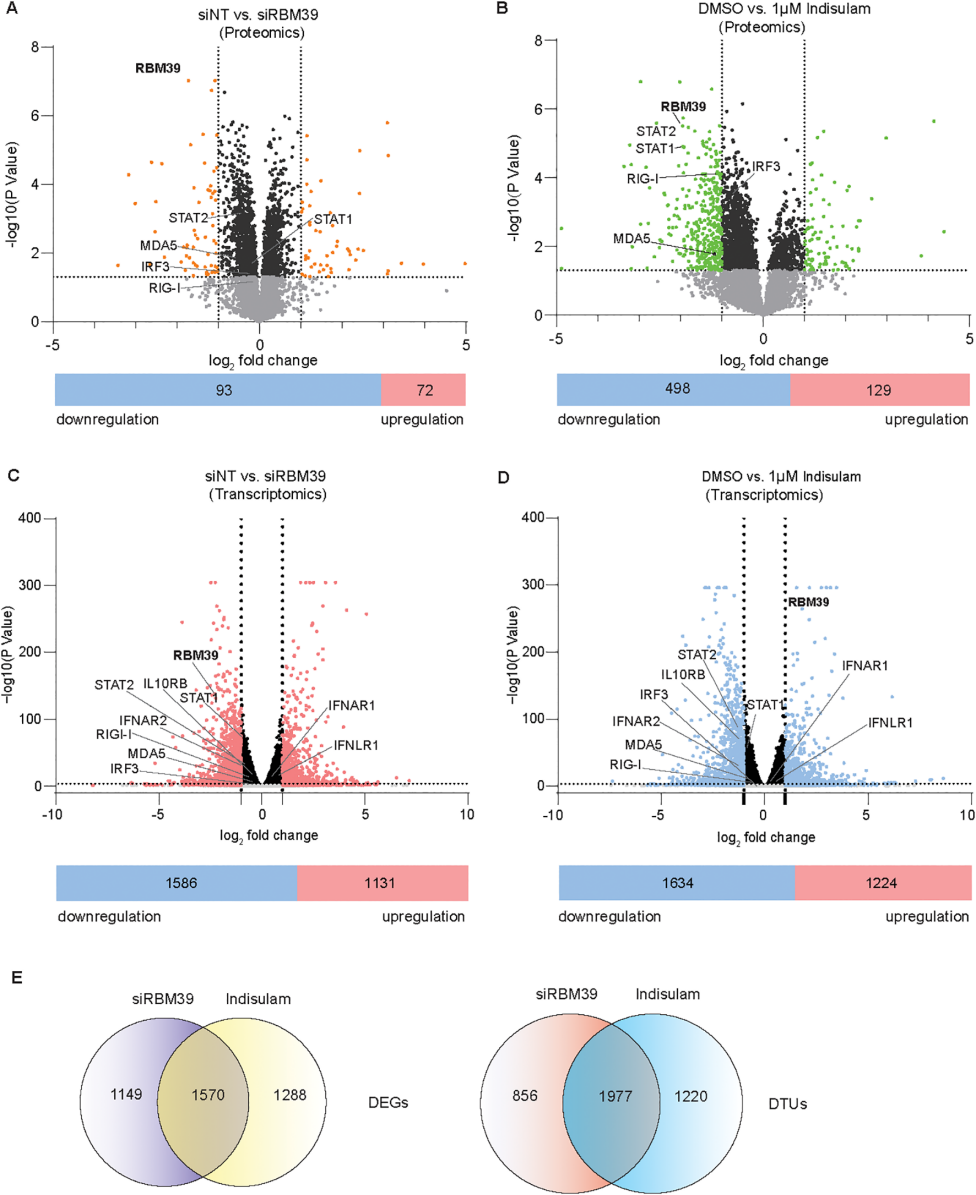
Global proteomic, transcriptomic, and splicing analysis upon modulation of RBM39 abundance. PH5CH cells were treated with siRBM39 vs. siNT or 1 µM indisulam vs. DMSO after 48 h. The proteome was measured by mass spectrometry. Transcriptome and splicing were measured through RNA-sequencing. **(A, B)** Volcano plot of the proteomic data for RBM39 knockdown **(A)** or indisulam-treated **(B)** samples; the numbers of down- and upregulated factors are shown under the plot. Factors with a fold change >2 or <0.5 and a *p*-value <0.05 were highlighted with orange **(A)** or green **(B)**. All factors with a *p*-value <0.05 but lower than twofold change are shown in black. IRF3, RBM39, RIG-I, MDA5, STAT1, and STAT2 were highlighted. **(C, D)** Volcano plot of DEGs in siRBM39- **(C)** and indisulam-treated **(D)** samples; the numbers of down- and upregulated factors are shown under the plot. DEGs with a fold change >2 or <0.5 and a *p*-value <0.05 were highlighted with red **(C)** and blue **(D)**. All factors with a *p*-value <0.05 but lower than twofold change are shown in black. IRF3, RBM39, RIG-I, MDA5, STAT1, STAT2, IFNAR1, IFNAR2, IFNLR1, and IL10RB were highlighted. **(E)** RNA-seq data is presented as differential expressed gene (DEG) and different transcript usage (DTU). The overview of DEG and DTU numbers in each condition and overlaps are indicated. The RNA-seq data are from three biological replicates (*n* = 3), and the proteomic data are from four replicates (*n* = 4). The proteomic data was analyzed via Perseus1.6.15.0. DEG and DTU analyses of individual genes were performed using DESeq2 and DRIMseq, respectively. Statistical significance was evaluated by “two-sample tests” (Student’s *t*-test with permutation-based FDR 0.05 and 250 number of randomizations) for proteomics and with the Wald test and corrected for multiple testing according to Benjamini–Hochberg for RNAseq analysis.

For the proteomic analysis, we found 165 proteins in *RBM39* knockdown samples and 627 proteins in indisulam-treated samples with significant changes (fold change <0.5 or >2, *p*-value <0.05) (**Figures 5A, B**; **Supplementary Table S2**). RBM39 was among the most significantly changed proteins, as expected (**Figure 5A**; **Supplementary Figure S5B**), whereas GAPDH signals were not significantly changed (**Supplementary Figure S5A**). The IRF3 levels were significantly reduced in both conditions, even though the changes upon *RBM39* knockdown were mild (**Figures 5A, B**; **Supplementary Figure S5C**). The generally lower magnitude of effects upon *RBM39* knockdown might be mediated by the difference in the timeframe of RBM39 reduction, acting faster due to direct protein degradation by indisulam treatment compared to the time required for siRNA-mediated reduction of RBM39 abundance. Importantly, we identified additional factors critically contributing to the cell intrinsic innate immune response, including RIG-I, MDA5, STAT1, and STAT2 (**Figures 5A, B**; **Supplementary Figures S5D–G**). Similar to IRF3, all of these factors showed significant changes in both conditions, with strong reduction in indisulam-treated samples and mild decline in *RBM39* knockdown samples, except for RIG-I that only was significantly decreased in indisulam-treated samples (**Supplementary Figure S5E**).

Due to the complex, multifactorial mode of action of RBM39 in transcription and splicing, we analyzed the RNAseq dataset in the context of differentially expressed genes (DEGs) and differential transcript usages (DTUs), indicative of changes in splicing, comparing the knockdown of *RBM39* and indisulam treatment. Our analysis identified 4,007 DEGs (fold change <0.5 or >2, *p*-value <0.05) and 4,053 DTUs (*p*-value <0.05) in *RBM39* knockdown and indisulam-treated samples. A total of 1,570 DEGs and 1,977 DTUs overlapped in both conditions (39% and 49% of total events, respectively) (**Figures 5C–E**; **Supplementary Tables S3**, **S4**), arguing for the reliability of the analysis. Comparing with proteomic data, the DEG analysis revealed more factors with significant fold change and identified similar gene numbers and pathways as in previous transcriptomic studies, including metabolism and cell division (**Figures 5C, D**; **Supplementary Table S3**) (22, 24, 58, 59). Interestingly, *RBM39* RNA abundance was significantly reduced upon knockdown (**Figure 5C**), as expected, but strikingly increased upon indisulam treatment due to the autoregulation of *RBM39* mRNA levels by protein abundance (**Figure 5D**) (60). Importantly and in agreement with the proteomic data, we found significant changes in factors involved in the cell intrinsic innate immune response, namely, RIG-I, MDA5, STAT1, and STAT2 and, in addition, type I and type III IFN receptors.

In summary, proteomic and transcriptomic analysis consistently identified several key factors of the interferon pathway beyond IRF3 that were regulated by RBM39, along with multiple other cellular processes identified in previous studies.

### RBM39 governs the transcription and splicing of crucial innate immune factors

Next, we aimed at elucidating whether the reduction of mRNA and protein abundance of the key factors of the IFN-response by RBM39 was mediated by alternative splicing or transcriptional regulation.

**Figure 6:**
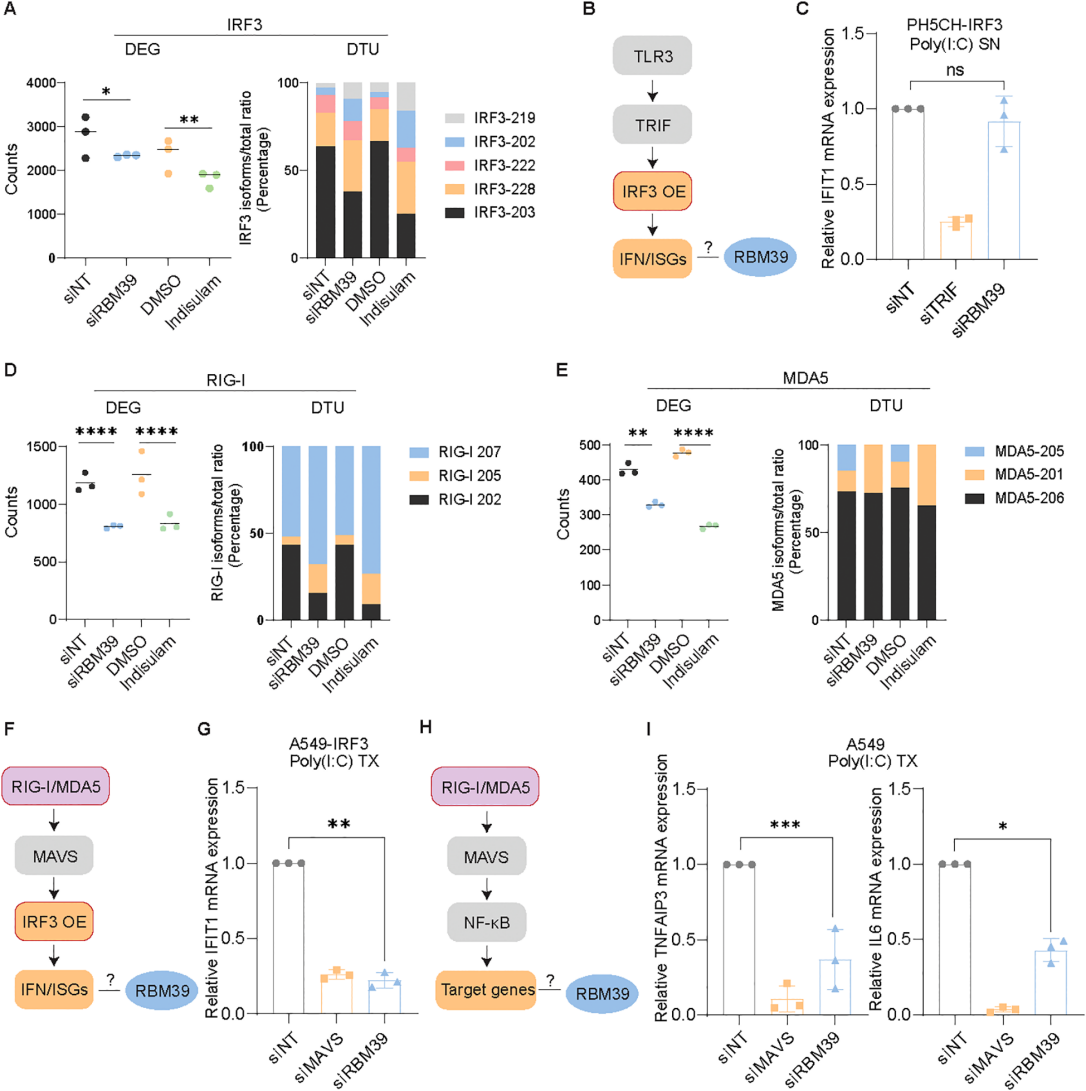
RBM39 controls the basal expression of *IRF3* and *RIG-I* and *MDA5.***(A)** DEG and DTU analysis of *IRF3* mRNA. **(B)** Simplified schematic of the TLR3 pathway. **(C)** IRF3 was ectopically expressed in PH5CH IRF3 KO cells, and cells were transfected with siRBM39 or siNT/siTRIF. At 48 h after knockdown, the cells were fed with 50 µg/mL poly(I:C) in the supernatant for 6 h. *IFIT1* mRNA expression levels were measured by RT-qPCR. **(D, E)** DEG and DTU analysis of *RIG-I***(D)** and *MDA5***(E)** mRNA. **(F)** Simplified schematic of RIG-I/MDA5-IRF3 signaling. **(G)** A549 cells overexpressing IRF3 were transfected with siRBM39 or siNT/siMAVS as controls for 48 h. After the knockdown, the cells were transfected with 0.5 µg/mL poly(I:C) in the supernatant for 6 h. *IFIT1* mRNA expression levels were measured by RT-qPCR. **(H)** Simplified schematic of the RIG-I/MDA5-NF-κB pathway. **(I)** A549 cells were silenced with siRBM39 or siNT/siTRIF for 48 h and then transfected with 0.5 µg/mL poly(I:C) for 6 h. *TNFAIP3* and *IL6* mRNA expression levels were measured by RT-qPCR. The data shown are from three biological replicates (*n* = 3); error bars indicate SD. The statistical significance of RT-qPCR data was assessed through Welch’s unpaired *t*-test, and for the transcriptomics data it was calculated with the Wald test and corrected for multiple testing according to Benjamini–Hochberg. ns = not significant, * = p < 0.05, ** = p < 0.01, *** = p < 0.001, **** = p < 0.0001.

For IRF3, we found a significant but mild reduction of *IRF3* mRNA read counts upon knockdown of *RBM39* or indisulam treatment (**Figure 6A**, left), suggesting a transcriptional regulation. This was confirmed by analyzing the impact of *RBM39* knockdown on *IRF3* mRNA by RT-qPCR (**Supplementary Figure S6A**) and on the activity of the *IRF3* promoter (61) using luciferase reporter plasmids (**Supplementary Figures S6B, C**), both showing a similar magnitude of reduction. In contrast, DTU analysis revealed substantial, consistent changes in splicing of *IRF3* mRNA when RBM39 was suppressed either by siRNA or by indisulam treatment, with a significant decrease of the functional *IRF3-203* isoform and an increased abundance of the particular isoform *IRF3-228*, which has been reported to have an inhibitory effect on innate immune activation (62) (**Figure 6A**, right panel). This data was validated upon *RBM39* knockdown via RT-qPCR using primers specifically targeting each of the *IRF3* isoforms that were detected in RNAseq, obtaining very similar results (**Supplementary Figure S6D**). To exclude the contribution of additional factors in the TLR3 pathway potentially affected by RBM39, we expressed IRF3 ectopically in PH5CH IRF3 KO cells. Indeed the overexpression of IRF3 restored the *IFIT1* mRNA induction upon RBM39 knockdown and TLR3 stimulation (**Figures 6B, C**), suggesting that IRF3 was the sole determinant of the TLR3 response regulated by RBM39.

In addition to IRF3, the cytosolic dsRNA sensors RIG-I and MDA5 were found to be significantly downregulated in the proteomic and transcriptomic analysis (**Figure 5**; **Supplementary Figure S5**). DEG/DTU analysis revealed that *RIG-I* expression was modulated both by transcription and splicing regulation, with the latter resulting in a lower abundance of the functional isoform 202 (**Figure 6D**), whereas *MDA5* expression was mainly decreased at the level of mRNA abundance (**Figure 6E**). To verify the consequence of RBM39 regulation on the RIG-I/MDA5 pathways beyond IRF3 (**Figure 6F**), we overexpressed IRF3 in A549 cells, thereby excluding any contribution of TLR3. In contrast to the TLR3 pathway in PH5CH cells, IRF3 expression did not suffice to restore *IFIT1* mRNA induction upon *RBM39* knockdown and poly(I:C) transfection (**Figure 6G**), indicating additional contributions of RIG-I and MDA5. Consequently, induction of the NF-kB-specific genes *TNFAIP3* and *IL6* was significantly reduced upon *RBM39* knockdown, when the pathway was triggered via RIG-I/MDA5 (**Figures 6H, I**).

Taken together, we found that the basal expression of IRF3 and RIG-I/MDA5 was regulated by the abundance of RBM39, with a significant impact on the induction of cell intrinsic innate immune responses.

### RBM39 regulates the IFN-JAK-STAT pathway

Our proteomic and transcriptomic analysis further identified components of the IFN-JAK-STAT pathway being regulated by RBM39, namely, STAT1 and 2 and type I and III IFN receptors; the latter were not detectable by proteomics likely due to low protein abundance. We therefore aimed to understand the mode of regulation and the functional relevance for the IFN response.

**Figure 7:**
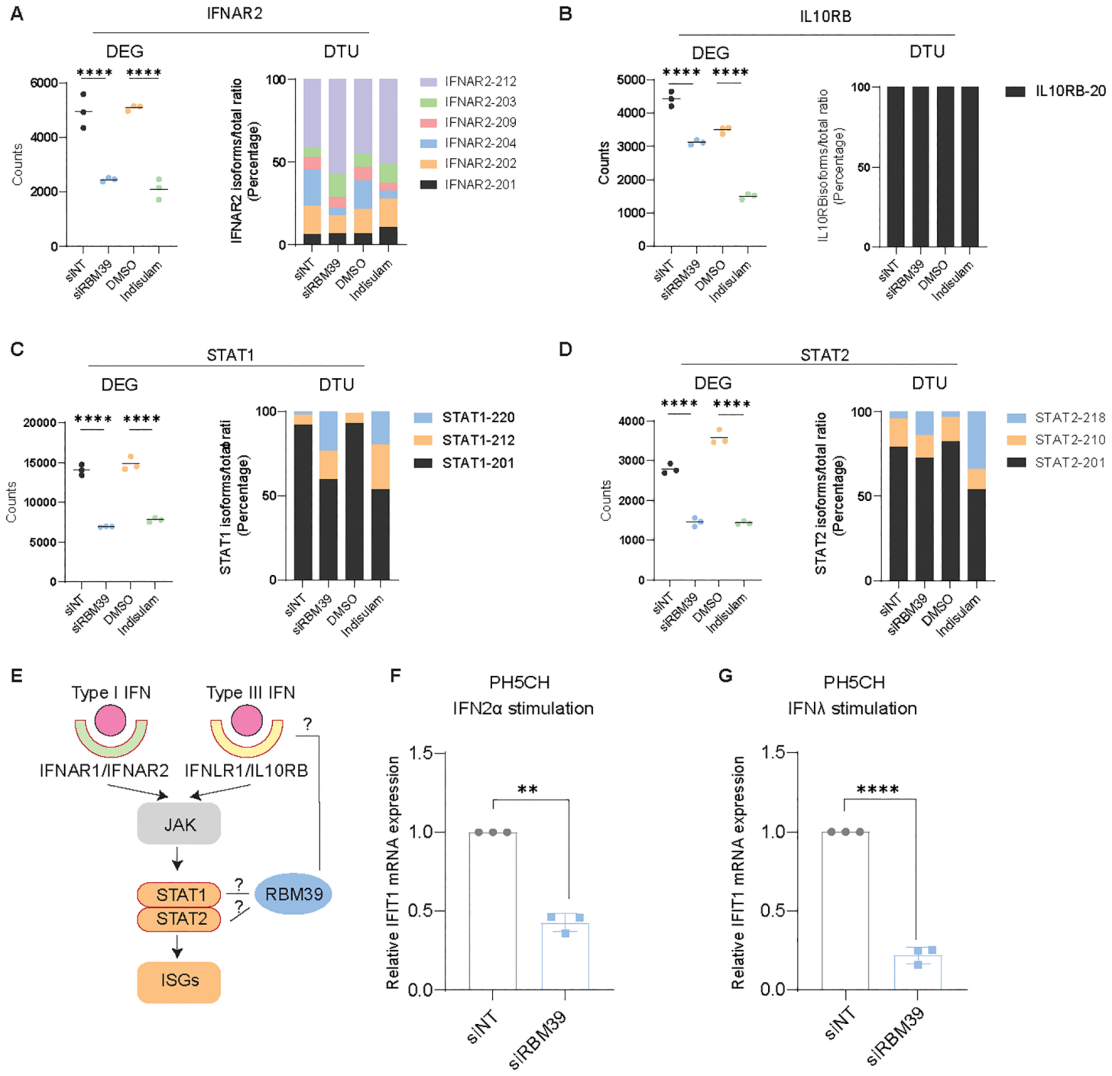
RBM39 impacts on IFN-JAK-STAT signaling. **(A–D)** DEG and DTU analysis of *IFNAR2***(A)**, *IL10RB***(B)**, *STAT1***(C)**, and *STAT2***(D)** mRNA. **(E)** Simplified schematic of the type I and type III IFN pathways. **(F, G)** PH5CH cells were transfected with siRBM39 or siNT as control for 48 h and then stimulated with IFNα2 **(F)** or IFNλ1 **(G)** for 24 h. *IFIT1* mRNA was measured by qPCR. The relative expression to siNT control is shown. mRNA fold change was normalized on *GAPDH*. The data were obtained from three biological replicates (*n* = 3); error bars refer to SD. The statistical significance of RT-qPCR data was assessed through Welch’s unpaired *t*-test. Transcriptomics data was calculated with the Wald test and corrected for multiple testing according to Benjamini–Hochberg. ** = p < 0.01, **** = p < 0.0001.

The receptor for type I IFNs is a heterodimer consisting of IFNAR1 and IFNAR2. While *IFNAR1* expression was upregulated only on the level of gene expression (**Supplementary Figure S6E**), *IFNAR2* mRNA abundance was reduced and splicing was modulated upon *RBM39* knockdown and indisulam treatment (**Figure 7A**). The type III interferon receptor is a heterodimer as well, composed of IFNLR1 and interleukin 10 receptor subunit beta (IL10RB), and also here the expression of both subunits was regulated by RBM39 in opposite directions, with an increase for *IFNLR1* (**Supplementary Figure S6F**) and a decrease for *IL10RB* (**Figure 7B**) RNA amounts, with minor or undetectable changes in the splicing pattern, respectively.

STAT1 and STAT2, forming heterodimers upon activation of the type I and type III IFN responses, were, in contrast, both reduced in protein expression levels upon depletion of RBM39 (**Supplementary Figures S5F,G**), mediated by changes in RNA abundance and associated with modified splicing patterns (**Figures 7C, D**).

To investigate the cumulative effect of up- or downregulation of these factors on the respective IFN response pathways (**Figure 7E**), we silenced RBM39 in PH5CH cells and stimulated them with IFNα2 and IFNλ1 to activate type I and type III IFN pathways, respectively. Consequently, the type I and type III IFN responses were both strongly impaired as indicated by reduced IFIT1 induction (**Figures 7F, G**), suggesting that RBM39 also regulates IFN-JAK-STAT signaling.

All in all, our RNA-seq and proteome analysis identified key factors of the IFN response affected by RBM39, such as IRF3, RIG-I, MDA5, STAT1, and STAT2 and type I/III receptors, arguing for a crucial role of RBM39 in the regulation of innate immunity.

## Discussion

Genome-wide CRISPR/Cas9 screens present a powerful tool to study host factors of innate immunity mostly in immune cells, such as macrophages (63), dendritic cells (64), or in the more commonly used Hela (65) and HEK 293T cells (66). In our study, we applied this approach in two liver cell lines, Huh7 and PH5CH, and specifically targeted the TLR3 pathway by poly(I:C) supernatant feeding. Eventually, we identified RBM39 as a novel factor that affects TLR3 signaling and has a broad impact on the cell intrinsic innate immune response by regulating the basal expression of IRF3 and other important pathway components.

Overall, most hit candidates identified in our screen turned out to be false positive due to several limitations. Firstly, many cells survived poly(I:C) induction despite the presence of the IFIT1-tBid reporter for unknown reasons, and this could neither be changed by the use of alternative inducible promoters driving tBid expression nor by additional rounds of transduction of the lentiviral vector encoding the reporter to increase gene copy numbers (data not shown). Secondly, TLR3-mediated induction of ISGs should also induce IFN-β, which will induce ISG expression in a paracrine manner via the JAK-STAT pathway, which could induce apoptosis in cells even if the TLR3 pathway would have been efficiently blocked by a gRNA depleting an essential factor. These limitations might have caused pseudo-enrichment of many irrelevant gRNAs. Indeed while the initially list of approximately 2,000 enriched genes included important known factors like TLR3, TRIF, and UNC93B1, it also lacked obvious genes like IRF3. The fact that many genes were not consistently enriched in both cell lines and in the replicates further complicated candidate definition.

RBM39 is a factor involved in the splicing and transcriptional regulation of many genes, particularly contributing to tumorigenesis (21, 22, 67), cell cycle control (12, 20), and metabolism (20), but so far its role in innate immunity has not been shown. Paradoxically, a previous study indicated that porcine reproductive and respiratory syndrome virus (PRRSV) infection upregulated porcine RBM39 expression, resulting in the dephosphorylation of c-Jun and subsequent downregulation of AP-1 signaling (68). Additionally, studies have reported that some viruses regulate splicing factors like RBM10 and U5 spliceosomal RNA (snRNA) as an evasion strategy (69, 70). While RBM39 has previously been shown to interact with the viral NF-κB homolog v-Rel, inhibiting its activation (67), the interplay between RBM39 and other human host NF-κB members like p50 and p105 has not been explored. Here we identified the basal expression of *IRF3* as one of the key factors of cell intrinsic innate immune responses being modulated by the knockdown of *RBM39* and indisulam-mediated RBM39 degradation. This resulted in significantly impaired activation upon both dsRNA administration and virus infection. In addition, we identified additional pathway components being modulated by RBM39, including the PRRs RIG-I and MDA5, IL-10RB, IFNAR2, STAT1, and STAT2. These factors all contributed to the reduced activation of the interferon response. Overall, this is supposed to result in an increased level of viral replication. In our infection experiments, RBM39 depletion only slightly stimulated viral RNA abundance, most likely due to the limited timeframe, providing not sufficient time to establish more pronounced phenotypes.

The complexity of regulatory functions mediated by RBM39 might explain why other transcriptomic and proteomic analyses missed its role in innate immunity so far (22–24, 53, 58, 71, 72). Interestingly, a gene set enrichment analysis of our transcriptomic data using gene ontology (GO) terms resulted in comparable data as in previous studies, highlighting the functions of RBM39 in metabolic processes and cytoskeleton, whereas terms connected to immunity were not significantly enriched (**Supplementary Figures S7A, B**). In contrast, the same analysis using hallmark gene sets (73) identified several immunity-related terms as significantly enriched, including TNFα signaling via NF-κB, inflammatory response, and interferon alpha response, with the latter only upon RBM39 knockdown (**Supplementary Figure S7C**). This suggests that modulation of immunity is indeed an important yet overlooked role of RBM39. In the transcriptomic and splicing analysis, the knockdown of *RBM39* by siRNA and indisulam treatment showed a wide degree of overlap, arguing for the specificity of indisulam toward RBM39 in the liver-derived cell lines used in our study which is in agreement with previous studies (53, 74). Overall, the transcriptomic analysis in our hands was more prone to identify innate immune response factors regulated by RBM39. While we could demonstrate that changes in IRF3 as well as that RIG-I/MDA5 expression contributed to the induction of cell intrinsic innate immune response, the data were more complex in the case of IFN treatment. Type I and type III IFNs bind to different receptors but engage identical signaling pathways (75). We observed a reduced expression of *IL10RB*, *IFNAR2*, *STAT1*, and *STAT2* but an increased expression of *IFNAR1* and *IFNLR1*, all essential for signal transduction, with STAT1 and STAT2 being involved in the response pathway to both type I and type III IFNs (75). Importantly, we confirmed that the aforementioned changes of these factors in sum resulted in the attenuation of both IFN pathways. In addition, further systemic effects of RBM39 ablation on innate immune responses are possible due to the complex involvement of IL-10RB in several cytokine receptor complexes, responding not only to IFNλ but also to pro- and anti-inflammatory cytokines including IL-10, IL-22, and IL-28/29 (76, 77).

RBM39 depletion was shown to have a strong anti-proliferative impact, which might explain our failure to obtain *RBM39* knockout clones in PH5CH cells (data not shown). This growth inhibitory effect has put forward RBM39 as a promising target for cancer therapy and contributed to numerous clinical trials implementing aryl sulfonamides like indisulam, albeit yet with limited success, likely due to the lack of predictive markers (78). In contrast, indisulam showed high activity in preclinical models like mice engrafted with human tumors (79) and, more recently, in neuroblastoma models (24, 79), associated with limited toxicity. Indisulam treatment of all human hepatic cells tested in our study, including two cancer cell lines (HepG2 and Huh7), one non-neoplastic cell line (PH5CH), and PHH, resulted in a significant degradation of RBM39, albeit at varying doses. Although RBM39 protein expression was similar in all of these cell lines, PHH could be less sensitive to indisulam due to lower *DCAF15* expression levels, critical for its mode of action. Importantly, RBM39 degradation was associated with a certain degree of cytotoxicity in all of our cell culture models. In contrast, indisulam treatment of PMH had no impact on RBM39 and IRF3 expression and lacked obvious cytotoxicity. The different indisulam sensitivity of PHH and PMH therefore imposes particular caution in interpreting toxicity data obtained in animal models. This is consistent with data showing only a minor effect of indisulam treatment on RBM39 expression in different organs of mice, including the liver, with significant changes only in murine kidney and the engrafted human tumors (23, 59, 79).

The lack of indisulam activity in PMH further precluded *in vivo* testing regarding immunosuppressive activities *in vivo*. Currently available aryl sulfonamides might generally not be suitable for clinical use as immunosuppressors due to their focus on anti-cancer activity. However, substances targeting RBM39 to proteasomal degradation based on alternative E3 ligases beyond DCAF15 might be envisaged as well as alternative tailored depletion strategies like molecular glues and proteolysis targeting chimeras (PROTACs) (80). In light of our data defining RBM39 as a regulator of key components of cell intrinsic innate immunity, it could be regarded as a candidate target to mitigate excessive innate immune responses contributing to viral pathogenesis (81) or autoimmune diseases (82).

## Data Availability

The datasets presented in this study can be found in online repositories. The names of the repository/repositories and accession number(s) can be found below: https://www.ncbi.nlm.nih.gov/, PRJNA1028350.
