# Correction: RBM39 shapes innate immunity by controlling expression of key factors of the interferon response

**DOI:** 10.3389/fimmu.2026.1782019

**Published:** 2026-01-26

**Authors:** Teng-Feng Li, Paul Rothhaar, Arthur Lang, Oliver Grünvogel, Ombretta Colasanti, Santa Mariela Olivera Ugarte, Jannik Traut, Antonio Piras, Nelson Acosta-Rivero, Vladimir Gonçalves Magalhães, Emely Springer, Andreas Betz, Hao-En Huang, Jeongbin Park, Ruiyue Qiu, Gnimah Eva Gnouamozi, Ann-Kathrin Mehnert, Viet Loan Dao Thi, Stephan Urban, Martina Muckenthaler, Matthias Schlesner, Dirk Wohlleber, Marco Binder, Ralf Bartenschlager, Andreas Pichlmair, Volker Lohmann

**Affiliations:** 1 Department of Infectious Diseases, Molecular Virology, Section Virus-Host-Interactions, Medical Faculty Heidelberg, Heidelberg University, Heidelberg, Germany; 2 Institute of Virology, School of Medicine, Technical University of Munich, Munich, Germany; 3 Department of Infectious Diseases, Molecular Virology, Medical Faculty Heidelberg, Heidelberg University, Heidelberg, Germany; 4 Division of Virus-Associated Carcinogenesis, German Cancer Research Center (DKFZ), Heidelberg, Germany; 5 Institute of Molecular Immunology, University Hospital Klinikum rechts der Isar, Technical University of Munich, Munich, Germany; 6 Bioinformatics and Omics Data Analysis, German Cancer Research Center (DKFZ), Heidelberg, Germany; 7 Faculty of Biosciences, Heidelberg University, Heidelberg, Germany; 8 School of Biomedical Convergence Engineering, Pusan National University, Yangsan, Republic of Korea; 9 Heidelberg University, Medical Faculty, Department of Pediatric Oncology, Hematology, Immunology and Pneumology, Heidelberg, Germany; 10 Department of Infectious Diseases, Virology, Medical Faculty Heidelberg, Heidelberg University, Heidelberg, Germany; 11 German Center for Infection Research (DZIF), Heidelberg Partner Site, Heidelberg, Germany; 12 Biomedical Informatics, Data Mining and Data Analytics, Faculty of Applied Computer Science and Medical Faculty, University of Augsburg, Augsburg, Germany; 13 German Center for Infection Research (DZIF), Munich Partner Site, Munich, Germany

**Keywords:** RBM39, IRF3, STAT1, STAT2, IFNs, splicing

Affiliations “Faculty of Biosciences, Heidelberg University, Heidelberg, Germany” and “School of Biomedical Convergence Engineering, Pusan National University, Yangsan, Republic of Korea” were omitted from the paper. These affiliations have now been added for author Jeonbin Park.

The original version of this article has been updated.

