# Supplementary material for: RBM39 shapes innate immunity by controlling the expression of key factors of the interferon response
Source: Front Immunol. 2025 Apr 22;16:1568056. doi: 10.3389/fimmu.2025.1568056 (PMC12054253; doi:10.3389/fimmu.2025.1568056)
Supplement: Supplementary file 1 [file DataSheet1.docx]

**Supplementary Materials**

**This PDF includes:**

Supplementary Fig. S1-7

Other Supplementary Material for this manuscript includes the following:

Table S1 to S5


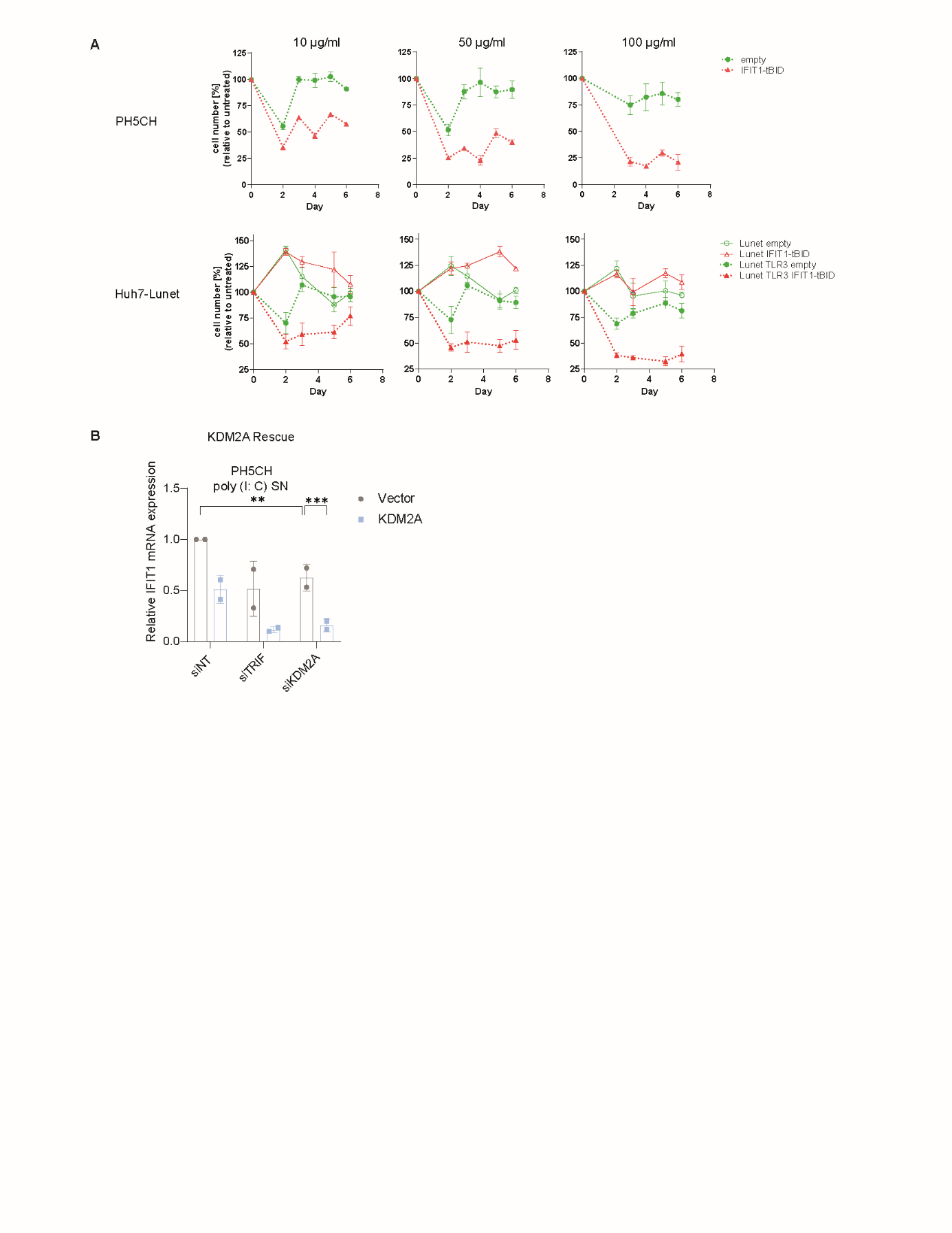


**Fig. S1.** **Efficiency of the tBID death reporter and the rescue of KDM2A.**

(**A**) PH5CH cells (upper panel) or Huh7-Lunet cells, with or without ectopic TLR3 (lower panel), stably expressing IFIT1-tBID or empty-tBID death reporter as control were supernatant-fed with the indicated concentrations of poly(I:C), or a same volume of PBS, for the indicated time. Cell viability was determined at the indicated time points using the WST-1 assay. Data are from four independent biological replicates (n=4). (**B**) PH5CH expressing an empty vector or *KDM2A* were transfected with the indicated siRNAs for 48 h. Cells were subsequently stimulated through poly(I:C) supernatant feeding. *IFIT1* mRNA was measured by qPCR. Data are from two biological replicates (n=2), error bars represent SD.


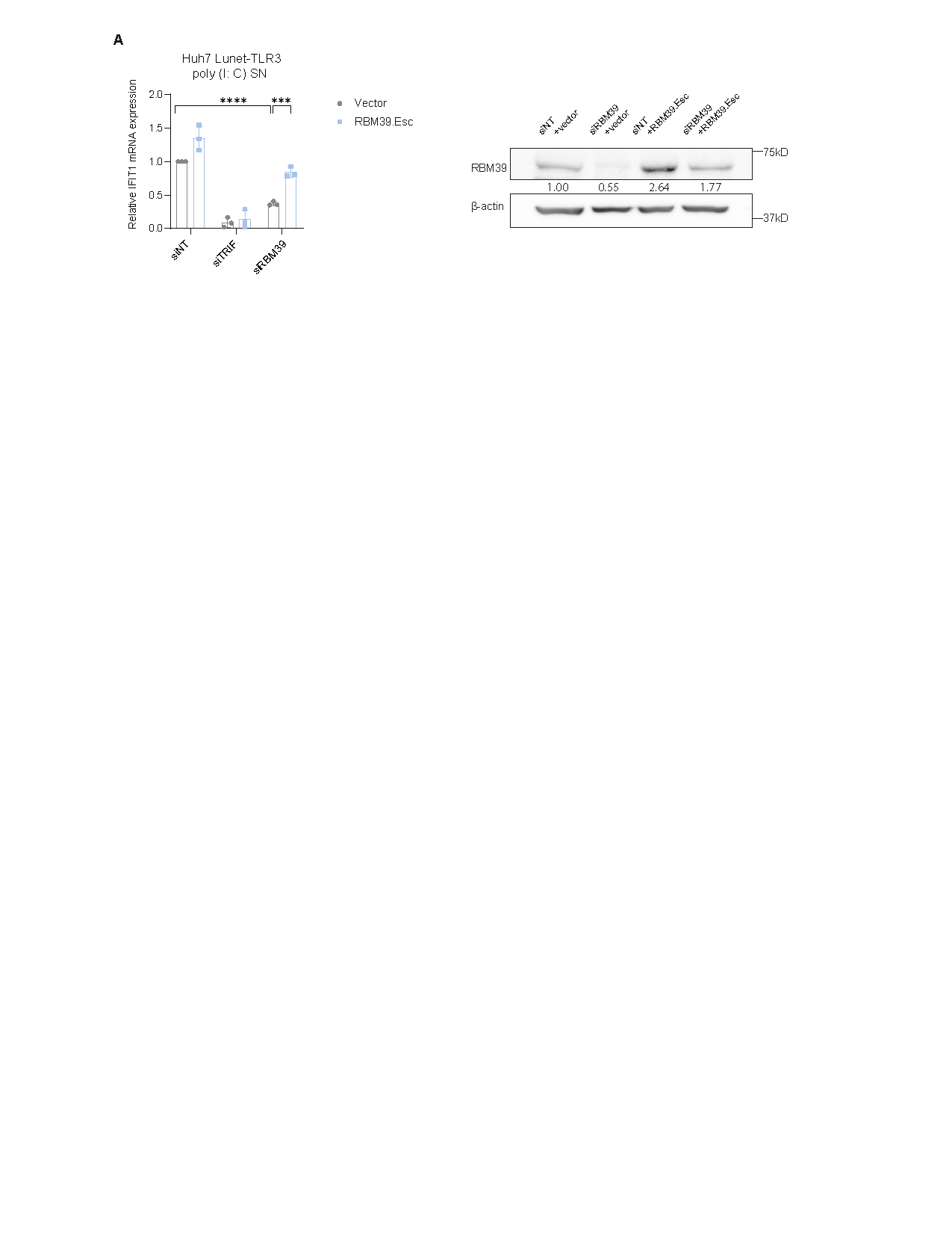


**Fig. S2. Rescue of TLR3 response by ectopic RBM39 expression in Huh7 Lunet-TLR3 cells.**

Huh7 Lunet-TLR3 cells expressing RBM39.Esc or empty vector were transfected with siRBM39 or siNT/siTRIF as controls for 48 h and then fed with 50 µg/ml poly(I: C) in the supernatant for 6 h. *IFIT1* mRNA was measured by RT-qPCR (left), RBM39 expression was measured by western blot (right). Data are from three biological replicates (n = 3), error bars refer to mean ± SD. Statistical significance was assessed through Welch’s unpaired *t*-test.


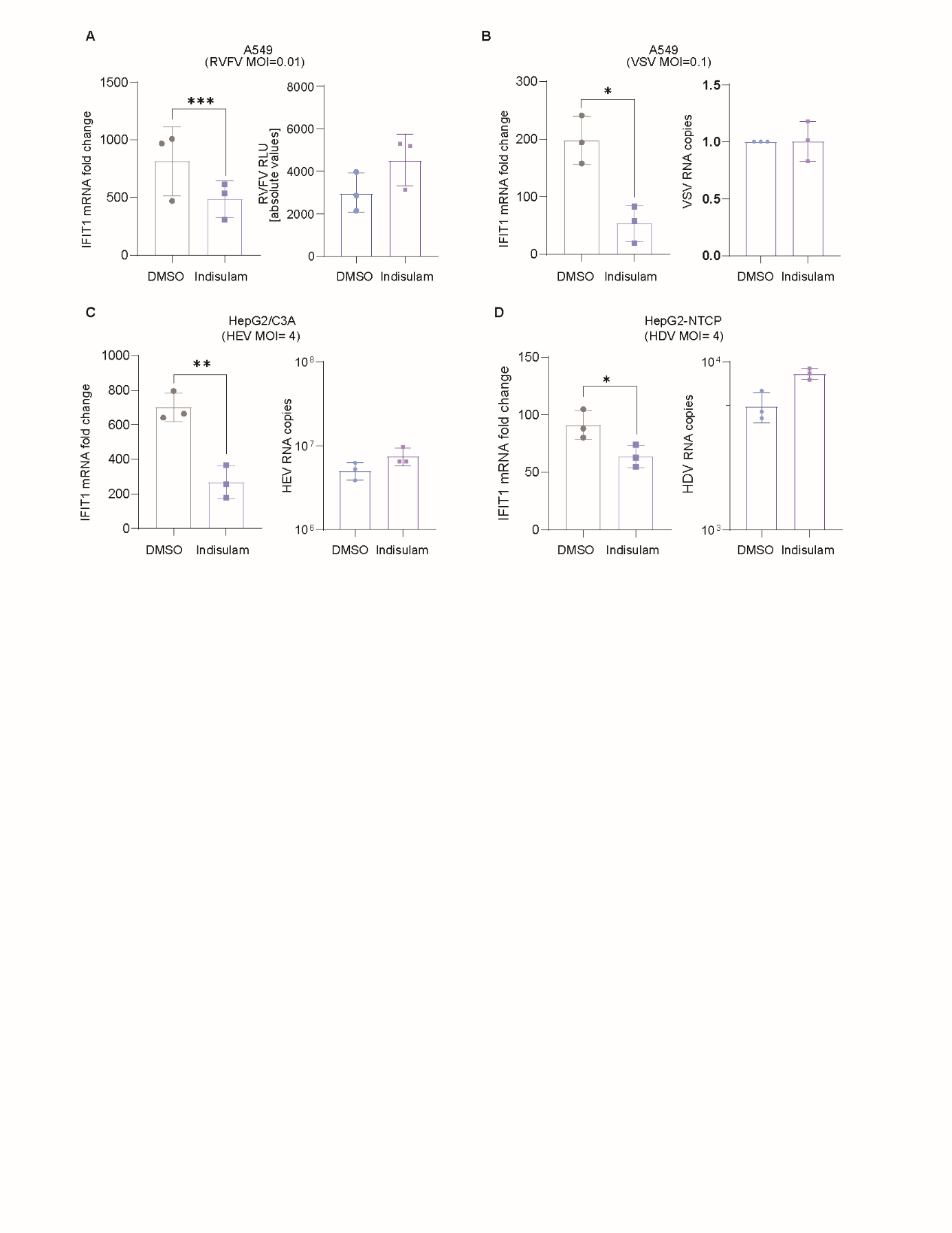


**Fig. S3. Indisulam treatment in different virus infection models.**

(**A**-**D**) A549 cells (**A** and **B**), HepG2/C3A (**C**) cells and HepG3-NTCP cells (**D**) were pre-treated with Indisulam or same amounts of DMSO as control for 48 h, and subsequently infected with Luc-RVFV (MOI= 0.01) for 24 h (**A**), rVSV-G (MOI=0.1) for 24 h (**B**), HEV (MOI= 4) (**C**) or HDV (MOI=4) (**D**) for 5 days, respectively. *IFIT1* mRNA (left) and viral mRNA (right) were measured by qPCR. mRNA fold change was normalized on *GAPDH*. Data are derived from three biological replicates (n = 3), error bars refer to SD. Statistical significance was assessed through Welch’s unpaired *t*-test.


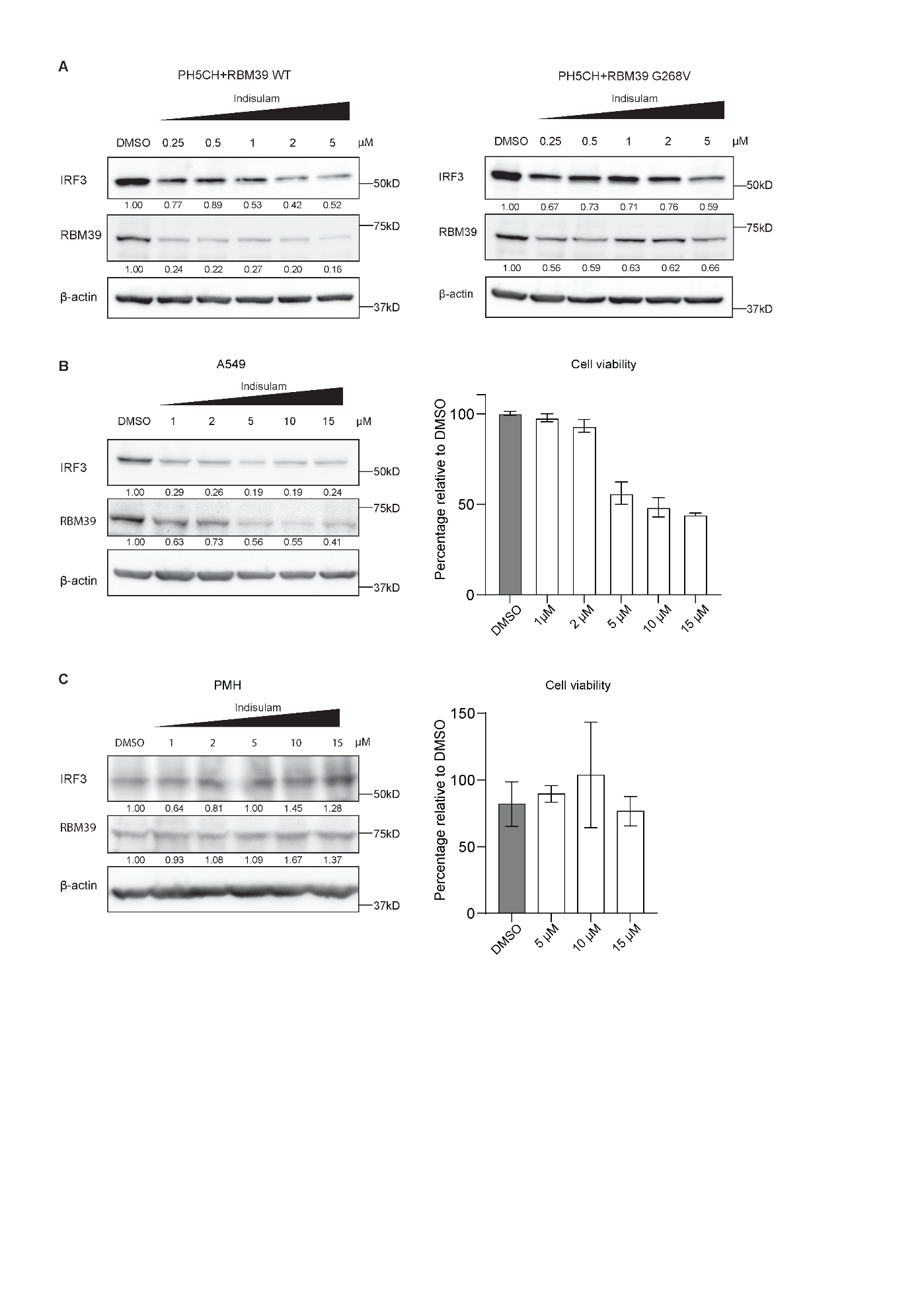


**Fig. S4.** **Impact of Indisulam-mediated RBM39 degradation on IRF3 in different cell lines.**

(**A** to **D**) PH5CH-RBM39 and PH5CH-RBM39 G268V cells (**A**), A549 cells (**B**) and primary mouse hepatocyte (PMH) (**C**) were treated with Indisulam at the indicated concentrations for 48 h. IRF3, RBM39 and β-actin protein expression levels were measured by western blot. Quantification of three independent experiments is shown as average number under the respective bands (left). Cell viability was measured via CellTiter-Glo luminescent cell viability assay (**B** and **C**, right). Data are from three biological replicates (n = 3), error bars indicate SD.


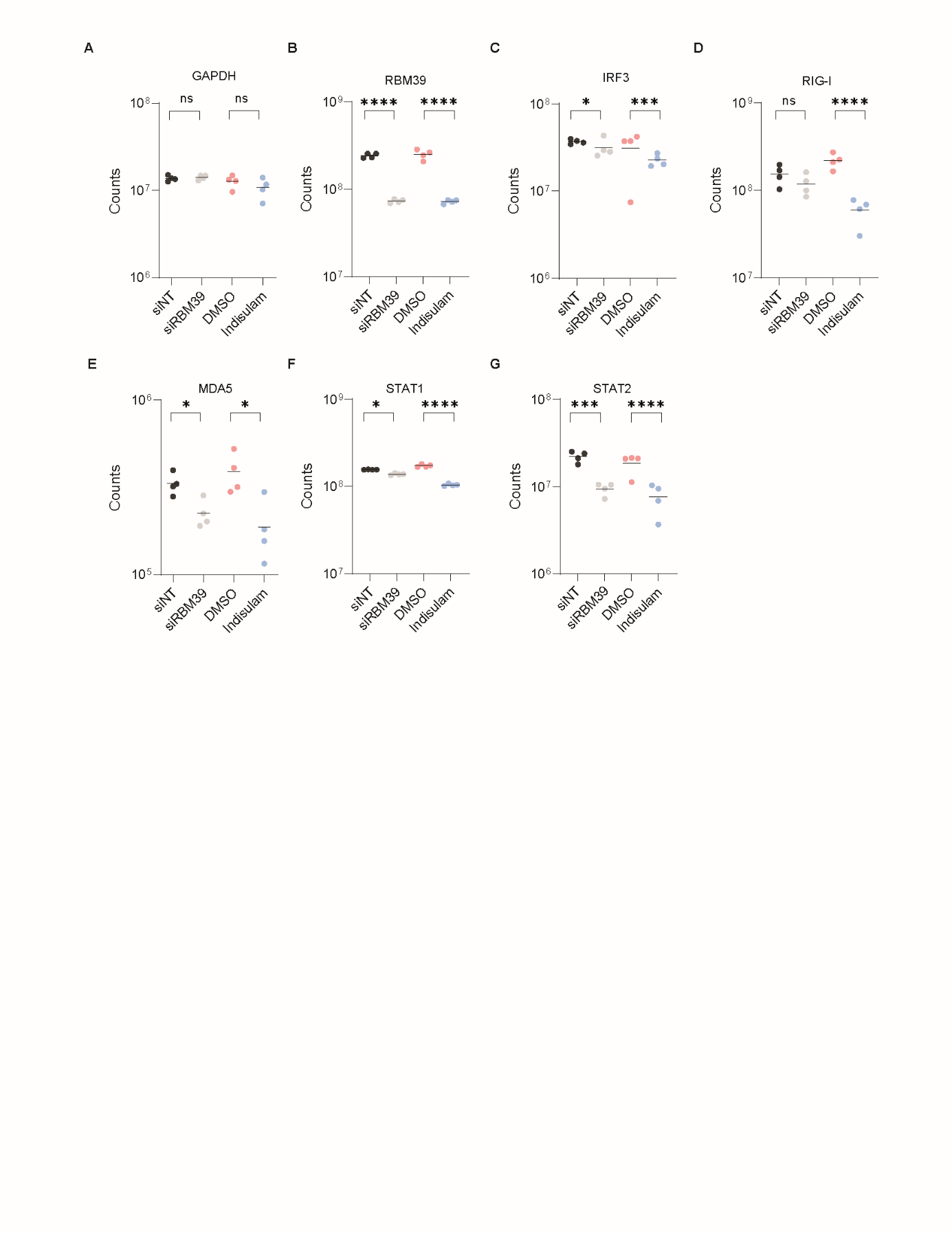


**Fig. S5.** **Analysis of individual protein expression levels from proteomics data.**

The protein level of GAPDH (**A**), RBM39 (**B**), IRF3 (**C**), RIG-I (**D**), MDA5 (**E**), STAT1 (**F**) and STAT2 (**G**) in siRBM39/siNT or Indisulam/DMSO-treated PH5CH samples. Data are derived from four biological replicates (n = 4), error bars refer to SD. Statistical significance was assessed by "two-sample tests" (Student’s t-test with permutation-based FDR 0.05 and 250 number of randomizations).


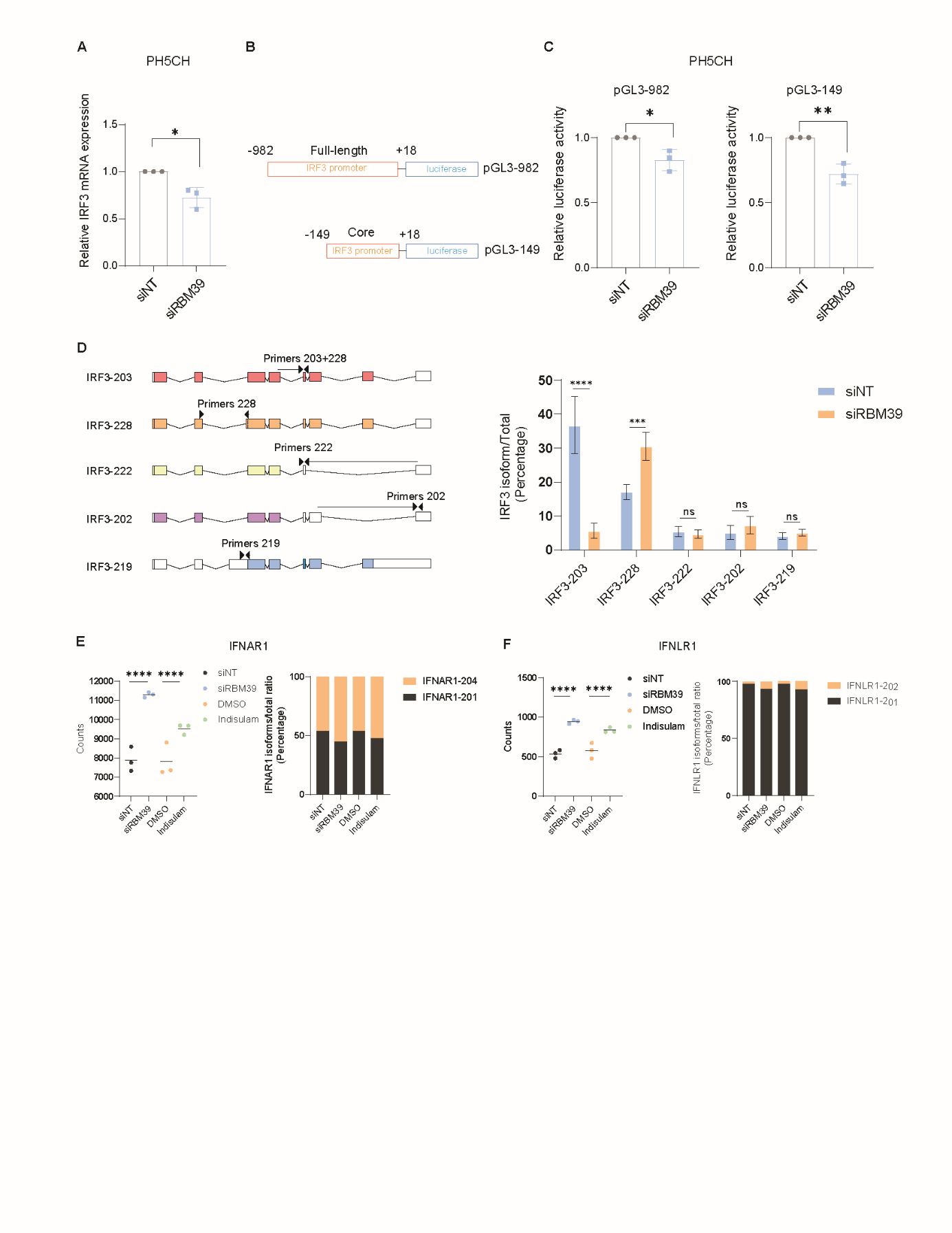


**Fig. S6.** **The transcription and splicing of *IRF3*, *IFNAR1* and *IFNLR1 mRNA***

(**A**) PH5CH cells were transfected with siRBM39 or siNT as controls. 48 h after transfection, *IRF3* and *RBM39* mRNA were measured by RT-qPCR. (**B**) Schematic of the firefly luciferase reporter harboring the full-length IRF3 promoter (pGL3-982-firefly) or core IRF3 promoter (pGL3-149-firefly). (**C**) PH5CH cells were transfected with pGL3-982-firefly or pGL3-149-firefly, pGL3-CMV-Gaussia was used as reference. Luciferase activity of firefly luciferase was normalized on that of Gaussia luciferase. Relative luciferase activity (Relative Light Units, RLU) is shown. (**D**) Schematic of *IRF3* isoforms (left). Specific primers targeting different isoforms are shown as arrows. White boxes indicate untranslated exons. *IRF3* isoforms/total *IRF3* ratio in PH5CH cells transfected with siRBM39 or siNT as control were identified by RT-qPCR. Note that isoform IRF3-228 only differs from -203 by insertion of an additional small exon, therefore no specific primers can be designed for -203. (**E** and **F**) DEG and DTU analysis of *IFNAR1* (**E**) and *IFNLR1* (**F**) mRNA. DEG and DTU analysis of individual genes were performed using DESeq2 and DRIMseq, respectively. Data shown are from three biological replicates (n = 3), error bars indicate SD. Statistical significance was assessed through Welch’s unpaired t test. Transcriptomics data was evaluated with the Wald test and corrected for multiple testing according to Benjamini-Hochberg.


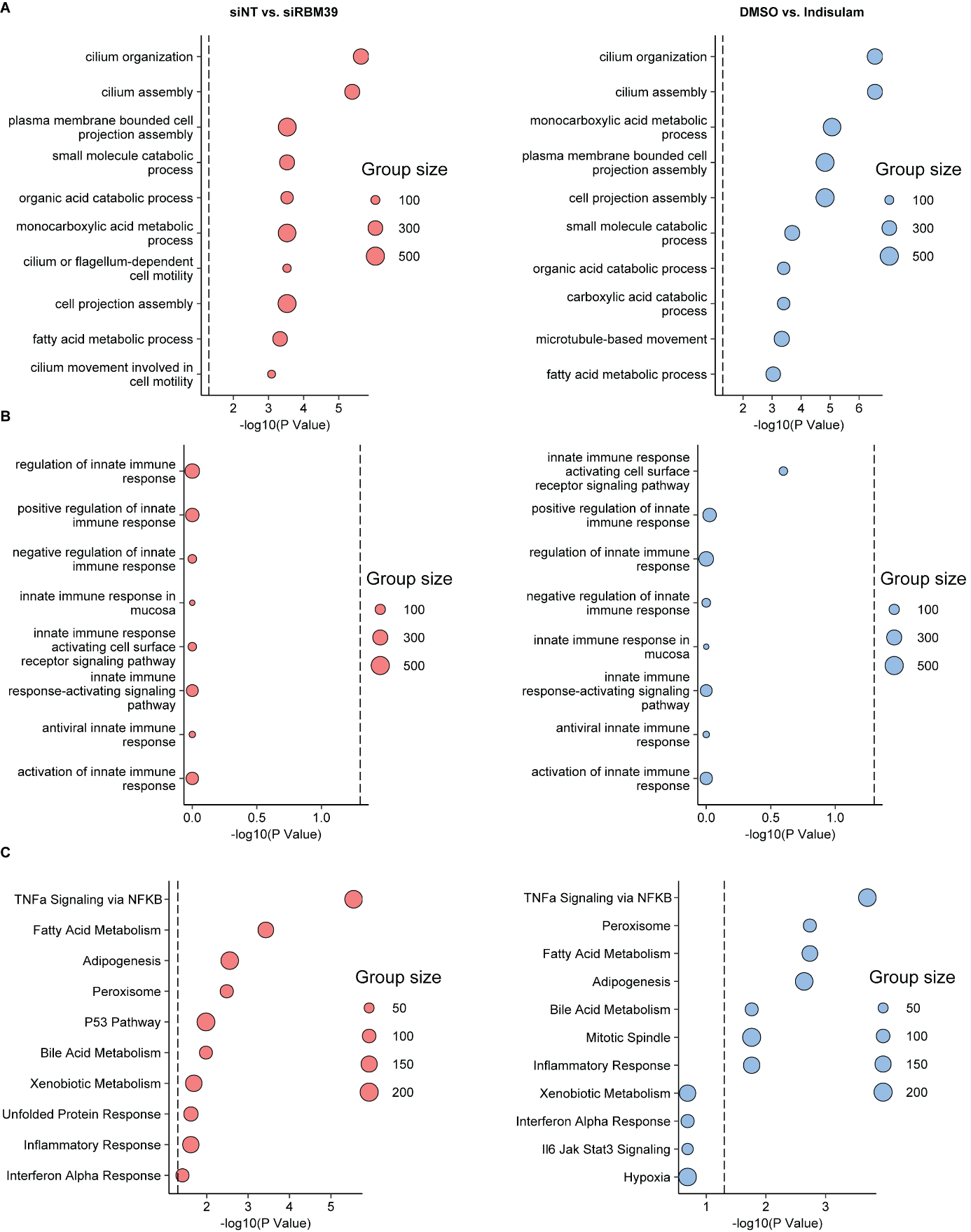


**Fig. S7. Gene set enrichment analyses.**

(**A-C**) Analyses based on the foldchanges in gene expression determined through RNAseq when comparing siRBM39 vs. siNT transfected cells (left panels) or Indisulam vs. DMSO treated cells (right panels). The dotted line indicates the threshold for statistical significance (p = 0.05). (**A**) Gene Ontology analysis for biological processes, depicted are the 10 terms with the most significant enrichment. (**B**) Gene Ontology analysis for biological processes, depicted are all terms containing the words “innate immune response”. (**C**) Hallmark analysis, depicted are the 10 pathways with the most significant enrichment. Statistical significance was determined using a permutation based test corrected for multiple testing according to Benjamini-Hochberg.
